# Multi-omics insights into growth impairment mechanisms in children with persistent diarrhea

**DOI:** 10.1128/spectrum.03905-25

**Published:** 2026-09-17

**Authors:** Jian Shen, Junle Yan, Ying Yuan, Lilin Le, Juan Xu, Bailu Chen, Xiaoying Liu, Huijie Chen, Lijun Chen, Meixiang Yi, Jiajia Lyu, Jun Diao, Xinlin Zhang, Yinqiu Zhao, Jingru Chen

**Affiliations:** 1 Department of Pediatrics, Yueyang Hospital of Integrated Traditional Chinese and Western Medicine, Shanghai University of Traditional Chinese Medicine 66322, Shanghai, China; Indian Institute of Technology (BHU) Varanasi 79203, Varanasi, Uttar Pradesh, India

**Keywords:** persistent diarrhea in children, stunting, butyrate, short-chain fatty acids, T helper 17 cells/regulatory T-cell balance, growth hormone-insulin-like growth factor 1 axis, multi-omics, machine learning

## Abstract

This study aimed to elucidate the key mechanisms underlying short stature with pediatric persistent diarrhea in children (PDC) and to propose an integrated model linking the butyrate-producing gut microbial niche, short-chain fatty acids (SCFAs), Th17/Treg balance, the growth hormone-insulin-like growth factor 1 (GH-IGF-1) axis, and growth regulation. Samples from healthy controls (HCs), PDC patients without short stature (PDC-NS), and PDC patients with short stature (PDC-S) were analyzed using multi-omics profiling and machine learning-based predictive modeling. The results showed that PDC-S patients exhibited disruption of core butyrate-producing bacterial communities and related metabolic pathways, markedly reduced fecal butyrate levels, systemic Th17/Treg imbalance characterized by a pro-inflammatory state, and dual suppression of receptor- and ligand-level components of the GH-IGF-1 axis. Multi-omics machine learning identified a five-factor risk prediction panel composed of metabolic, immune, and endocrine markers, while causal inference further established butyrate as a central regulator of growth. Mouse experiments further validated that combined intervention with butyrate, *Faecalibacterium prausnitzii*, and recombinant human growth hormone (rhGH) ameliorated growth retardation. Overall, this study proposes a precision therapeutic strategy based on butyrate and GH co-intervention, providing new mechanistic insights and translational tools for PDC-associated short stature.

**IMPORTANCE:**

The research conducted in this study holds significant implications for improving the understanding and treatment of stunted growth with persistent diarrhea in children (PDC). By unraveling the intricate pathways linking gut microbiota, immune responses, and growth hormone regulation, the study sheds light on the underlying mechanisms contributing to stunting in these vulnerable populations. The identification of key factors, such as butyrate-producing bacteria and the Th17/Treg balance, not only enhances our comprehension of PDC-related growth impairment but also offers a promising avenue for targeted interventions. The proposed integrated mechanistic model and intervention strategy pave the way for precision therapies tailored to address the specific biological mechanisms at play, potentially leading to more effective and personalized treatments for children suffering from PDC-associated stunting.

## INTRODUCTION

Diarrheal diseases remain one of the leading causes of morbidity and mortality among children under 5 years of age worldwide (1). Among these, persistent diarrhea in children (PDC) is particularly severe, as its duration exceeds 14 days and frequently results in chronic malnutrition and developmental impairment, posing a major public health challenge in low- and middle-income countries (2). PDC not only induces acute weight loss but also exerts long-term effects on children’s linear growth and has been closely associated with adult stunting, immune dysfunction, and an increased risk of chronic metabolic diseases (3–5). Stunting represents the most prominent phenotypic manifestation of childhood malnutrition, closely linked to early-life infectious exposures, intestinal dysfunction, and immune imbalance (6). However, despite clinical evidence demonstrating the association between PDC and impaired growth, the molecular mechanisms and systemic biological networks underlying this relationship remain incompletely understood. In particular, the synergistic interplay among post-infectious chronic inflammation, nutrient malabsorption, and endocrine suppression has yet to be fully elucidated, leaving a substantial gap in current research (7, 8).

The gut microbiota, often regarded as an essential “invisible organ” of the host, plays a pivotal role in child growth and development (9, 10). Under physiological conditions, the gut microbiota ferments dietary fibers to produce short-chain fatty acids (SCFAs)—including acetate, propionate, and butyrate. Among these, butyrate not only serves as a primary energy source for intestinal epithelial cells but also regulates immune responses, maintains intestinal barrier integrity, and modulates host metabolic networks, thereby directly contributing to growth regulation (11–13). Evidence indicates that chronic diarrhea is closely associated with gut microbiota dysbiosis. Disruption of the intestinal microecology may be accompanied by the loss of core butyrate-producing genera, such as *Faecalibacterium*, reduced short-chain fatty acid metabolic capacity, and impaired intestinal mucosal immune homeostasis (14, 15). Furthermore, SCFAs deficiency may impair the homeostatic regulation of regulatory T cells (Treg), thereby promoting a proinflammatory Th17 response and exacerbating chronic inflammation (16, 17). Nevertheless, most of these findings remain at the level of microbiota-metabolite associations. A systematic mechanistic elucidation and causal validation of how SCFAs modulate both immune and endocrine systems to influence the short stature phenotype following PDC are still lacking.

Existing evidence suggests that SCFAs play a crucial regulatory role in the crosstalk between the immune and endocrine systems (18, 19). SCFAs such as butyrate and propionate can activate G protein-coupled receptors (GPCRs) (GPR41 and GPR43) and inhibit histone deacetylases (HDACs), thereby promoting Treg differentiation and suppressing proinflammatory Th17 responses, ultimately alleviating chronic inflammation (20–22). Disruption of immune homeostasis is not confined to the intestinal mucosa; rather, it can exert systemic effects through cytokine- and chemokine-mediated signaling, leading to dysregulation of the growth hormone-insulin-like growth factor 1 (GH-IGF-1) axis, which, in turn, impairs skeletal growth and chondrocyte differentiation (23, 24). However, existing studies remain largely restricted to single-layer observations or isolated pathways, and there is still a lack of a verifiable, integrated mechanistic framework linking the gut microbiota-SCFA axis with immune regulation and endocrine growth signaling. Consequently, the causal chain and cross-system regulatory interactions have yet to be systematically elucidated.

Artificial intelligence-driven multi-omics integration technologies have opened new avenues for deciphering complex molecular networks (25, 26). By integrating gut microbiota sequencing, metabolomics, immune transcriptomics, and hormonomics data sets, it becomes possible to delineate multi-pathway and multi-factor interaction networks at a systems level. However, traditional statistical methods often struggle to effectively handle high-dimensional data, noise, and batch effects (27). Machine learning (ML) approaches, such as random forest regression, XGBoost, and Elastic Net, can identify important variables from high-dimensional features and substantially improve predictive performance. Interpretable algorithms such as SHAP can further help identify core bacterial genera or metabolic pathways associated with target phenotypes, as well as their nonlinear effect thresholds (28, 29). In addition, topological analysis of multi-omics heterogeneous networks and ablation studies, combined with causal inference methods based on propensity scores and inverse probability of treatment weighting (IPTW), have the potential to model molecular network propagation and rigorously identify true causal chains (25, 26). The integration of these techniques is expected to overcome the limitations of conventional single-feature analyses and provide a panoramic systems-level understanding of the biological mechanisms underlying PDC-associated short stature.

Based on the above evidence, we proposed a central hypothesis: depletion of core butyrate-producing gut microbial communities and the resulting deficiency of SCFAs, particularly butyrate, disrupt systemic immune homeostasis, especially by inducing a pro-inflammatory Th17/Treg imbalance. This imbalance subsequently suppresses the GH-IGF-1 axis through a specific gut-immune-endocrine cascade, ultimately driving the growth-retardation phenotype in PDC. To test this hypothesis, we adopted a “computation-to-validation” research strategy. We hypothesized that integrating multi-omics data with machine learning and causal inference algorithms would enable the prioritization of key regulatory nodes from high-dimensional associations and support the construction of a streamlined predictive feature panel with strong clinical translational potential. Subsequently, these computational predictions, including specific microbial taxa, metabolites, and immune pathways identified as putative causal drivers, were translated into testable biological targets and systematically validated using mouse models and cell-based experiments. This approach aimed to confirm the mechanistic link between the predicted regulatory axis and growth impairment, thereby providing a solid theoretical basis for multi-target synergistic intervention in PDC-associated short stature.

## MATERIALS AND METHODS

### Study design and overall workflow

This study adopted a multi-level design centered on an in-house clinical cohort and further supported by animal and cell-based validation experiments. The aim was to systematically characterize the associations among gut microbiota, fecal short-chain fatty acids (SCFAs), the peripheral immune transcriptome, and the GH/IGF-1 growth axis in the phenotype of persistent diarrhea-associated short stature in children. In the clinical component, three pediatric cohorts were first established: healthy controls (HCs), children with persistent diarrhea without severe stunting (PDC-NS), and children with persistent diarrhea and severe stunting (PDC-S). Multi-omics data matrices were then constructed using fecal 16S rRNA amplicon sequencing, targeted SCFA metabolomics, peripheral blood mononuclear cell (PBMC) transcriptomics, and serum endocrine/inflammatory factor profiling. Finally, cross-omics correlation analysis, partial correlation analysis, machine learning modeling, network topology analysis, and propensity score-weighted analysis were performed to evaluate the relationship between the butyrate–immune–IGF-1 axis and pediatric growth phenotypes.

The in-house clinical cohort included children from the HC, PDC-NS, and PDC-S groups, with 150 participants in each group, and was used for baseline clinical characterization, serological measurements, and subsequent omics subsampling. A stratified sampling strategy was applied for omics assays. For 16S rRNA sequencing, 30 samples per group were originally planned; after filtering based on sequencing depth, sample completeness, and quality control criteria, 81 samples were ultimately included, comprising 29 HC, 27 PDC-NS, and 25 PDC-S samples. Targeted fecal metabolomics and PBMC transcriptomics were completed in 30 samples per group. All 16S rRNA sequencing, SCFA metabolomics, PBMC transcriptomics, and clinical phenotype data used for the core bioinformatics analyses were derived from clinical samples collected and assayed in this study. Public databases were not used as the primary source of analytical data and served only as potential independent literature support or external trend references; they were not included in the core statistical and modeling analyses presented in Fig. 10 to 16.

The primary outcome was defined as short stature, determined by a height-for-age *Z*-score (HAZ) < −2 according to the World Health Organization (WHO) Child Growth Standards. The PDC-S group was defined as children with persistent diarrhea and HAZ < −2, whereas the PDC-NS group was defined as children with persistent diarrhea but HAZ ≥ −2. The HC group consisted of healthy children without persistent diarrhea and with growth and developmental indicators within the normal range. Secondary outcomes included fecal concentrations of butyrate, acetate, propionate, and total SCFAs; serum levels of insulin-like growth factor 1 (IGF-1), insulin-like growth factor-binding protein 3 (IGFBP-3), and inflammatory cytokines; expression levels of immune-related genes such as FOXP3 and IL17A in peripheral blood mononuclear cells (PBMCs); and the relative abundances of key bacterial genera/species and PICRUSt2-predicted functional pathways.

### Study participants and clinical data collection

Children under 5 years of age were consecutively enrolled in this study. PDC was diagnosed based on the duration of clinical symptoms, stool characteristics, and physician-documented diagnosis records. At enrollment, clinical information was collected for all participants, including age, sex, height, weight, body mass index (BMI), recent infection history, antibiotic exposure, probiotic use, and nutritional status. Height and weight were measured by trained researchers using standardized instruments. Each child was measured at least twice; if the difference between the two measurements exceeded the predefined allowable error range, a third measurement was performed, and the average value was used.

The HAZ was calculated according to the WHO Child Growth Standards. A HAZ < −2 was defined as the short-stature phenotype. Nutritional status was determined based on age, sex, body weight, height, and clinical nutritional assessment results. Antibiotic exposure was defined as the use of systemic antibiotics within 4 weeks before sampling, whereas probiotic exposure was defined as the use of probiotic preparations within 2 weeks before sampling. These variables were included as potential confounders in subsequent partial correlation analyses, machine learning sensitivity analyses, and propensity score models.

### Sample collection, storage, and nucleic acid extraction

All samples were collected according to standardized operating procedures. Fresh fecal samples were collected into sterile cryopreservation tubes, immediately snap-frozen in liquid nitrogen, and transferred to a −80°C freezer within 30 min for long-term storage. Fecal samples were used for microbial DNA extraction and targeted SCFA metabolomic analysis. Peripheral venous blood was collected into EDTA anticoagulant tubes and procoagulant tubes. EDTA-anticoagulated blood was used for PBMC isolation and RNA extraction, whereas serum isolated from procoagulant blood after centrifugation was used for the measurement of IGF-1, IGFBP-3, and inflammatory cytokines.

Total microbial DNA was extracted from fecal samples using the QIAamp PowerFecal Pro DNA Kit (Qiagen, Germany). DNA concentration, purity, and integrity were assessed using a NanoDrop spectrophotometer and agarose gel electrophoresis. PBMCs were isolated using Ficoll-Paque density-gradient centrifugation, followed by total RNA extraction using TRIzol reagent (Invitrogen, USA). RNA samples were evaluated for OD_260/280_ ratios using NanoDrop, and RNA integrity was assessed using an Agilent 2100 Bioanalyzer. Samples meeting the required RNA integrity number (RIN) threshold were subjected to subsequent transcriptome library construction.

### 16S rRNA amplicon sequencing and microbiome data processing

16S rRNA amplicon sequencing was performed by amplifying the V3–V4 region of the bacterial 16S rRNA gene using the primers 341F (5′-CCTAYGGGRBGCASCAG-3′) and 806R (5′-GGACTACNNGGGTATCTAAT-3′). After purification, quantification, and library construction, the PCR products were subjected to paired-end sequencing on an Illumina sequencing platform.

Raw sequencing data were quality-controlled and analyzed using QIIME2 (30). Low-quality reads, adaptor sequences, and chimeric sequences were first removed. Sequence denoising was then performed using the DADA2 plugin to generate an amplicon sequence variant (ASV) feature table. Taxonomic annotation was conducted using the SILVA database (Release 138) and a pre-trained Naive Bayes classifier. Sample quality-control criteria included an effective sequence count of no fewer than 10,000 reads, unique matching between sample IDs and clinical information, and the absence of obvious sequencing contamination or abnormally low-complexity features. After quality control, 29 HC samples, 27 PDC-NS samples, and 25 PDC-S samples were ultimately included for 16S rRNA-related analyses.

Microbial α-diversity was assessed using the Shannon index, Chao1 index, and observed ASVs. Intergroup differences were evaluated using the Kruskal–Wallis test, followed by Dunn’s post hoc test when appropriate. β-Diversity was calculated based on the Bray-Curtis distance matrix, and principal coordinate analysis (PCoA) was used to visualize differences in community structure among groups. PERMANOVA with 999 permutations was applied to test overall differences in microbial community structure, while PERMDISP was used to assess within-group dispersion, thereby avoiding the misinterpretation of differences in dispersion as differences in community structure. Differential bacterial taxa were identified using LEfSe analysis, with the screening criteria set as LDA score > 4.0 and *P* < 0.05.

### Construction of ASV/OTU tables, taxonomic annotation, and genus-/species-level matrices

The ASV/OTU abundance table was organized with ASV IDs as rows and sample IDs as columns. The taxonomic annotation table included the Kingdom, Phylum, Class, Order, Family, Genus, and Species levels. To generate low-dimensional feature matrices suitable for machine learning and network analyses, the ASV abundance table was first merged with the taxonomy table according to ASV ID and then summarized at the genus and species levels, respectively. Whether unannotated taxa or taxa labeled as “Unassigned” were retained or excluded depended on the specific analytical purpose. All high-quality ASVs were retained for community structure analyses, whereas only taxa clearly annotated at the genus or species level were included in candidate microbial feature analyses.

Microbial abundance data were converted to relative abundance before differential analysis and machine learning modeling. For correlation and network analyses, centered log-ratio (CLR) transformation was applied to reduce compositional data bias, with a small pseudocount added before transformation to avoid zero-value issues.

### PICRUSt2 functional prediction and analysis of butyrate biosynthesis pathways

Microbial functional potential was predicted using PICRUSt2 based on the 16S ASV feature table and representative sequences. The MetaCyc pathway abundance table generated by PICRUSt2 was used for downstream functional pathway analyses. This study focused on pathways related to SCFA production, particularly butyrate biosynthesis pathways such as PWY-5022. The pathway abundance matrix was matched with the sample metadata using 16S sample IDs and subjected to group-based differential analysis.

For statistical analysis, pathway abundance values were first log-transformed or *Z*-score standardized. Comparisons among the three groups were performed using the Kruskal–Wallis test, followed by pairwise Wilcoxon rank-sum tests for post hoc comparisons. Multiple testing correction was conducted using the Benjamini-Hochberg method. The abundance of butyrate biosynthesis pathways was further included as a candidate feature in machine learning regression models to predict the absolute concentration of fecal butyrate.

### Targeted metabolomic detection and quantification of fecal SCFAs

Fecal SCFAs were absolutely quantified using gas chromatography-mass spectrometry (GC-MS). Approximately 50 mg of each freeze-dried fecal sample was weighed and mixed with acidified extraction solution containing an internal standard, followed by thorough vortexing and ultrasonic extraction. After high-speed centrifugation at 4°C, the supernatant was collected for GC-MS analysis. 2-Methylvaleric acid, or an equivalent stable internal standard suitable for SCFA quantification, was used as the internal standard.

The analytical platform consisted of an Agilent 7890B gas chromatography system coupled to a 5977B mass spectrometer. An HP-FFAP capillary column suitable for volatile fatty acid separation was used. Target metabolites, including acetate, propionate, butyrate, and isobutyrate, were absolutely quantified using calibration curves generated from authentic standards, and concentrations were expressed as μmol/g dry fecal weight. Blank samples, standards, and quality-control samples were included in each analytical batch. If the relative standard deviation (RSD) of the quality-control samples exceeded 15%, the analytical quality of that batch was re-evaluated.

In subsequent statistical analyses, butyrate was included as the core metabolite in the correlation analysis in Fig. 10, regression modeling in Fig. 11, classification modeling in Fig. 12, and propensity score analysis in Fig. 16. Total SCFAs were defined as the sum of acetate, propionate, and butyrate concentrations.

### PBMC transcriptome sequencing and immune gene expression analysis

Total RNA extracted from peripheral blood mononuclear cells (PBMCs) was used for mRNA library construction after passing quality control and was sequenced on the Illumina NovaSeq 6000 platform using a PE150 strategy. Raw reads were assessed for quality using FastQC, and low-quality reads and adaptor sequences were removed using Trimmomatic or equivalent tools. The quality-controlled reads were aligned to the human reference genome GRCh38, and gene expression quantification was performed using featureCounts (31).

The normalized gene expression matrix was used for differential expression analysis and extraction of target immune-related genes. Differential expression analysis was performed using DESeq2, with screening criteria set as |log_2_FoldChange| > 1 and FDR < 0.05. Immune-related analyses focused on genes associated with Treg/Th17 balance and inflammatory responses, including FOXP3, IL17A, RORC, IL10, TNF, and IL6. FOXP3 and IL17A were included as core immune indicators in the cross-omics analyses presented in Fig. 10 and 12.

### Serum IGF-1, IGFBP-3, and inflammatory cytokine measurements

Serum samples were separated by centrifugation and stored at −80°C until analysis. IGF-1, IGFBP-3, and inflammatory cytokines were quantified using commercial enzyme-linked immunosorbent assay (ELISA) kits or a clinical immunoassay platform. All assays were performed according to the manufacturers’ instructions, and actual concentrations were calculated based on standard curves. Replicate wells and quality-control samples were included in each experimental batch. Samples were re-assayed if the coefficient of variation between replicate wells exceeded the predefined threshold.

Serum IGF-1 was included as a key indicator of the GH/IGF-1 axis in butyrate correlation analysis, partial correlation analysis, classification modeling, and propensity score analysis. IGFBP-3 was used as an auxiliary growth-axis indicator for descriptive analyses and sensitivity analyses.

### Multi-omics sample matching and data matrix construction

All omics data were mapped using unified sample metadata. The metadata included the unified participant ID, 16S sequencing ID, fecal metabolomics ID, PBMC transcriptomics ID, clinical information ID, and group information. Cross-omics integrative analyses included only samples that could be accurately matched across multiple omics layers. The final core sample set used for the integrative analyses in Fig. 10 through 16 comprised 81 participants, corresponding to the 16S quality-controlled samples: 29 in the HC group, 27 in the PDC-NS group, and 25 in the PDC-S group.

Although fecal metabolomics and PBMC transcriptomics were completed in 30 samples per group, the 16S quality-controlled matched samples were used as the core data set for joint analyses with microbiome data. Single-omics analyses could still use the complete data set of 30 samples per group for each omics layer, whereas cross-omics correlation analyses, machine learning models, and network analyses were performed using the unified matched core sample set to ensure that variables from different omics layers were derived from the same participants.

Data preprocessing included missing-value inspection, outlier detection, unit harmonization, log transformation, and *Z*-score standardization. For machine learning models, all continuous features were standardized during model training. To prevent information leakage, feature selection and standardization procedures were embedded within the cross-validation workflow or performed only within the training set.

### Cross-omics correlation analysis

To evaluate the associations among gut microbiota, short-chain fatty acids (SCFAs), the immune transcriptome, and the GH/IGF-1 axis, Spearman rank correlation analysis was performed to calculate correlation coefficients between continuous variables. Variables included in the correlation analysis comprised fecal concentrations of butyrate, acetate, and propionate; the relative abundances of key butyrate-producing bacterial genera/species; the abundances of PICRUSt2-predicted butyrate biosynthesis pathways; the expression levels of immune-related genes in peripheral blood mononuclear cells (PBMCs), including FOXP3, IL17A, RORC, and IL10; and serum levels of IGF-1, IGFBP-3, and inflammatory cytokines.

To reduce the influence of potential confounders, including age, nutritional status, and antibiotic exposure, partial residual analysis was further performed to assess associations between variables after covariate adjustment. Specifically, linear models including covariates were constructed separately for the target variable and the explanatory variable. Model residuals were then extracted and subjected to Spearman correlation analysis, and partial residual scatter plots were generated for visualization. This approach helped avoid the problem of singular covariance matrices that may occur in conventional partial correlation analysis under small-sample conditions.

### Multi-omics machine learning modeling and robustness assessment

To evaluate the predictive capacity of multi-omics features for fecal butyrate levels and the risk of short stature in children, regression and classification models were constructed separately. Given the limited sample size of the cross-omics core data set in this study (*n* = 81), machine learning analyses did not use high-dimensional full-feature inputs. Instead, low-dimensional feature panels were constructed based on biological prior knowledge to reduce the risk of overfitting (Fig. S1).

Regression models were mainly used to predict the absolute concentration of fecal butyrate. Candidate features included the abundances of key butyrate-producing bacterial genera/species, PICRUSt2-predicted butyrate biosynthesis-related pathways, and other SCFA-related functional features. XGBoost regression (32) and random forest regression (33) were used for model construction, and model performance was evaluated using repeated cross-validation. Model performance metrics included the coefficient of determination (*R*^2^), mean squared error (MSE), and root mean squared error (RMSE). Feature importance was interpreted using SHAP (SHapley Additive exPlanations) (34), and only core features with high contribution and stable performance were presented.

Classification models were used to evaluate the ability of multi-omics features to discriminate the risk of PDC-S. Candidate features included key butyrate-producing bacteria, fecal butyrate concentration, serum IGF-1, and PBMC immune indicators such as FOXP3 and IL17A. Because HAZ is highly related to the disease definition, it was excluded from the main model to avoid information leakage. The primary classification model was Elastic Net logistic regression, which was compared with Ridge logistic regression and random forest.

Model training and evaluation were performed using repeated stratified cross-validation. Receiver operating characteristic (ROC) curves and precision–recall (PR) curves were calculated based on out-of-fold (OOF) predicted probabilities. Model performance was further assessed by calculating 95% confidence intervals using bootstrap resampling, and permutation tests were performed to evaluate whether the models showed obvious overfitting. Model calibration was assessed using calibration curves, and clinical net benefit was evaluated using decision curve analysis (DCA).

To improve model interpretability, the SHAP framework was used to quantify the contribution of each feature to model output. Considering that single train/test splits may produce inflated AUC values under small-sample conditions, the analysis did not rely solely on a single split result. Instead, model robustness was assessed using repeated cross-validation, bootstrap-based stability analysis, and the frequency with which features were repeatedly selected into the model.

### Cross-omics network analysis and hub node identification

A cross-omics correlation network integrating microbiota, metabolites, immune genes, and hormones was constructed using Spearman correlation analysis. Only edges meeting the criteria of |*ρ*| >0.30 and FDR < 0.05 were retained. Network nodes included key microbial taxa, SCFAs, immune-related genes, and serum hormone indicators.

Network construction and topological analysis were performed using the igraph package. Topological parameters, including degree centrality, betweenness centrality, and closeness centrality, were calculated. Nodes with high degree centrality were defined as potential hub nodes.

To assess the stability of hub nodes, bootstrap resampling was further performed 100 times to reconstruct the network repeatedly, and the frequency with which each node was identified as a hub across repeated networks was recorded. Hub nodes with high stability were considered to have strong topological importance across different sample subsets.

### Propensity score weighting and causal association analysis

To evaluate the independent associations of butyrate levels with IGF-1 and short-stature risk, inverse probability of treatment weighting (IPTW) analysis was performed. Participants were classified into high- and low-butyrate groups according to fecal butyrate concentrations, and a propensity score model was constructed using age, sex, nutritional status, antibiotic exposure, and other clinical covariates.

Inverse probability weights were calculated based on the propensity scores, and covariate balance was assessed using standardized mean differences (SMDs). An SMD < 0.1 after weighting was considered to indicate good covariate balance.

After IPTW adjustment, weighted linear regression was used to assess the association between butyrate levels and serum IGF-1, whereas weighted logistic regression was used to evaluate the association between butyrate levels and PDC-S risk. Adjusted odds ratios (ORs) and 95% confidence intervals were calculated. Subgroup analyses, placebo analysis, and assessment of propensity score overlap distributions were further performed to evaluate the robustness of the results.

### Animal experiments

All animal experiments were performed using specific-pathogen-free (SPF) C57BL/6J mice (3–4 weeks old, body weight 14–16 g, male-to-female ratio 1:1). Mice were housed under controlled environmental conditions (temperature: 22 ± 2°C; humidity: 50–60%; 12-h light/dark cycle). Furthermore, to ensure high scientific credibility, transparency, and reproducibility, all animal experiments, housing conditions, and endpoint evaluations were designed and reported in strict adherence to the Animal Research: Reporting of *In Vivo* Experiments (ARRIVE) guidelines 2.0. Upon arrival, mice underwent a 7-day acclimatization period before experimentation. To minimize potential bias, animals were stratified by sex and randomly assigned to experimental groups, with a minimum of 15 mice per group. The sample size was determined based on an a priori statistical power analysis using G*Power software (version 3.1.9). Assuming a large effect size (Cohen’s *d* = 1.2) for the primary phenotypic endpoints (e.g., growth plate thickness and serum IGF-1 levels) based on our preliminary data and previous literature, an alpha level of 0.05, and a statistical power of 80%, a minimum of 12 mice per group was calculated as necessary. We enrolled 15 mice per group to strictly account for potential attrition related to the DSS and caloric restriction induction protocol. To minimize biological variability and potential litter effects, strict age- and sex-matched littermates were randomly assigned to different groups. Furthermore, cages were systematically rotated within the housing racks weekly to mitigate environmental micro-variations.

A PDC-like phenotype was induced by the combined administration of low-dose Dextran sulfate sodium (DSS) (1–1.5%, provided in drinking water) and mild caloric restriction (10–15% reduction in total energy intake) to mimic the dual stress of chronic diarrhea and nutritional deficiency. The induction period lasted 14–21 days. Mice in the control group received an equivalent volume of sterile drinking water and were fed a complete standard diet. This vehicle-treated, time-matched control group was essential to establish the baseline trajectory of normal skeletal and endocrine maturation against which the PDC-induced stunting could be accurately quantified. Throughout the experimental period, health status was monitored daily, including body weight, food intake, and fecal characteristics, to ensure model stability and reproducibility.

### Intervention procedures

The selection of biological targets and experimental conditions for the *in vivo* interventions was directly informed by our multi-omics computational predictions. Specifically, the high feature importance of *Faecalibacterium prausnitzii* (*F. prausnitzii*) and butyrate in the LightGBM and SHAP analyses, combined with their hub status in the heterogeneous network (GCN), identified them as primary upstream therapeutic targets. Furthermore, the causal inference model (CausalForest) predicted a significant positive effect of butyrate on the GH-IGF-1 axis (ATE = +18.4 ng/mL per SD increase), which motivated the selection of SCFA/butyrate supplementation and recombinant human growth hormone (rhGH) as direct interventions to validate this quantitative prediction. After the PDC-like phenotype was stably established, mice were subjected to four categories of interventions: (i) SCFA supplementation: acetate, propionate, or butyrate (administered individually or in optimized combinations) were delivered via drinking water or oral gavage at a dose of 150–300 mg/kg body weight, based on previously validated protocols from the literature. (ii) Key microbial supplementation: live cultures of *Faecalibacterium prausnitzii* (*F. prausnitzii*) or *Blautia* spp. were prepared under anaerobic conditions at a final concentration of 10^9^ CFU/mL and administered daily by oral gavage. (iii) GH supplementation: recombinant human growth hormone (rhGH) was subcutaneously injected daily at a dose of 0.5–1 mg/kg, dissolved in sterile saline. Injection sites were alternated to prevent local tissue irritation. (iv) Immune modulation: low-dose butyrate was added to the diet or drinking water to enhance Treg function, or alternatively, a small-molecule inhibitor (SR1001) was administered to selectively suppress Th17 activation. All interventions were conducted under blinded conditions, and investigators responsible for outcome assessment were unaware of group assignments to eliminate observer bias.

### Endpoint evaluation and biomarker assessment

At the end of the intervention period, comprehensive endpoint assessments were performed, encompassing phenotypic, histological, molecular, and metabolic parameters. Phenotypic evaluation included measurements of body weight, body length (nose-to-anus length), and tibial growth plate thickness. The latter was determined by hematoxylin-eosin (H&E) staining of paraffin-embedded sections, followed by microscopic quantification. Serum GH and IGF-1 levels were measured by CLIA, and hepatic IGF-1 mRNA expression was quantified by RT-qPCR with GAPDH as the internal control. Protein expression levels of IGF-1 were further assessed by Western blot analysis.

Fecal SCFA concentrations were analyzed using GC-MS on an Agilent 8890 GC system coupled to a 5977B mass detector. Data were processed and quantified using the internal standard method to ensure analytical accuracy. Intestinal barrier integrity was comprehensively evaluated by multiple indicators, including immunohistochemical (IHC) detection of tight junction protein ZO-1 and mucin MUC2 expression, as well as measurement of serum fluorescence intensity following oral administration of fluorescein isothiocyanate (FITC)-dextran (excitation/emission wavelengths: 485 nm/535 nm). Immune cell profiling was conducted using a BD FACSCanto flow cytometer to quantify the proportions of CD4^+^CD25^+^ Forkhead Box P3 (FoxP3)^+^ regulatory T (Treg) cells and IL-17A^+^ T helper 17 cells (Th17). Data acquisition and analysis were performed with FlowJo software (version 10).

All experimental data were independently reviewed and verified by two researchers before being entered into a centralized database, and then integrated and cross-validated with the multi-omics analysis results. Statistical analyses were performed using R software (version 4.3.1). Normality tests were conducted prior to hypothesis testing, and multiple comparison corrections were applied using the Benjamini-Hochberg procedure to control the FDR < 0.05, ensuring the robustness and reliability of the findings.

### Cell experiments

This study employed multiple human- and mouse-derived immune and epithelial cell systems to systematically evaluate the roles of SCFAs and their associated signaling pathways in immune regulation and hormonal axis function. PBMCs were isolated from healthy volunteers and patient samples using Ficoll-Paque density gradient centrifugation. Mouse splenocytes were obtained by mechanical homogenization followed by erythrocyte lysis, yielding single-cell suspensions. HepG2 cells (human hepatocellular carcinoma cell line) were cultured in Dulbecco’s modified Eagle medium (DMEM) supplemented with 10% fetal bovine serum (FBS), whereas Caco-2 cells (human colonic epithelial cell line) were maintained in high-glucose DMEM containing 20% FBS. All cultures were maintained at 37°C in a humidified atmosphere with 5% CO_2_. Primary CD4^+^ T cells and dendritic cells (DCs) were purified from PBMCs using magnetic-activated cell sorting (MACS) technology. Cell purity exceeded 95%, as confirmed by flow cytometry.

Cells were exposed to graded concentrations of SCFAs (0.5, 1, 2.5, and 5 mM) to simulate physiological and high-dose conditions. SCFA stock solutions were freshly prepared under sterile conditions and used immediately to ensure chemical stability. To verify receptor-dependent mechanisms, experiments incorporated GPR41/43 antagonists (e.g., GLPG0974) and agonists (e.g., AR420626). In parallel, HDAC inhibitors, such as Trichostatin A (TSA), and their corresponding activators were applied to mimic the epigenetic regulatory effects of SCFAs. In addition, key gut commensal bacteria, including *F. prausnitzii* and *Blautia* spp., were anaerobically cultured, and their cell-free fermentation supernatants were collected after filtration through 0.22 μm membranes for subsequent cell treatment. All experiments included vehicle controls, and cell morphology and viability were continuously monitored throughout the incubation period, with cell viability quantified using the CCK-8 assay.

Each experimental condition was performed with at least three independent biological replicates, and each experiment included three technical replicates to ensure reproducibility. The immunophenotype of PBMCs and T cells was analyzed by flow cytometry to determine the proportions of Tregs (CD4^+^CD25^+^FoxP3^+^) and Th17 (CD4^+^IL-17A^+^). IGF-1 secretion from HepG2 cells was quantified using an ELISA. The barrier integrity of Caco-2 cells was evaluated by measuring transepithelial electrical resistance (TEER) and FITC-dextran permeability. The maturation and antigen-presenting functions of DCs were assessed by flow cytometry for surface molecules including CD80, CD86, and MHC-II, and cytokine secretion profiles were analyzed using a Luminex multiplex assay. All raw data were documented in laboratory notebooks and synchronously uploaded to the electronic data management system, ensuring traceability and consistency for subsequent multi-omics integration and causal inference analyses.

### Statistical analysis

Statistical analyses were independently conducted in both R (version 4.3.1) and Python (version 3.10) environments, and key results were cross-validated across the two platforms to ensure accuracy and reproducibility. Prior to analysis, data integrity was assessed, and outliers were identified using boxplots and *Z*-score methods. The distribution of continuous variables was assessed using the Shapiro-Wilk test for normality. For normally distributed data, two-sample *t*-tests or one-way/multifactor analysis of variance (ANOVA) were applied to evaluate intergroup differences. Post hoc comparisons among multiple groups were performed using either the Tukey’s honest significant difference (HSD) test or the Dunnett test. For non-normally distributed data, the Mann-Whitney *U*-test (for two-group comparisons) and the Kruskal–Wallis test (for multiple groups) were used, followed by Dunn’s multiple-comparison test when applicable. Categorical variables were compared using either the chi-square test or Fisher’s exact test, depending on sample distribution and expected counts. To control the FDR arising from multiple hypothesis testing, all *P* values were adjusted using the Benjamini-Hochberg correction, with FDR < 0.05 considered statistically significant.

Correlations between continuous variables were calculated using the Spearman rank correlation coefficient, and partial correlations were applied when necessary to control for potential confounding covariates. For cross-omics correlations involving microbial abundance, metabolite concentrations, and gene expression levels, data were centered log-ratio (CLR) transformed to mitigate the influence of compositional bias inherent in microbiome data. Microbial diversity analyses were performed using the phyloseq and vegan R packages. Alpha diversity was evaluated by calculating the Shannon, Simpson, and Chao1 indices. Beta diversity was assessed using Bray-Curtis dissimilarity and weighted and unweighted UniFrac distances. Intergroup differences in community structure were tested by permutational multivariate analysis of variance (PERMANOVA) (adonis, 999 permutations), while within-group dispersion was examined using permutational analysis of multivariate dispersions (PERMDISP) to rule out false positives due to variance heterogeneity.

Missing data were addressed using multiple imputation by chained equations (MICE) implemented in the mice R package, with 20 imputations (*m* = 20) and 50 iterations per imputation. The imputation model incorporated all key analytical variables and covariates to improve the accuracy and stability of the imputed data sets. To assess the robustness of the findings, sensitivity analyses were performed by comparing results from the imputed data sets with those from complete-case analyses. All statistical tests were two-tailed, and statistical significance was defined as either *P* < 0.05 or FDR < 0.05 after multiple comparison correction. Figures and plots were generated using ggplot2, matplotlib, and seaborn, ensuring clarity, consistency, and compatibility with varying resolution and publication formats.

## RESULTS

### Baseline matching, multi-omics data completeness, and batch-effect correction

**Fig 1:**
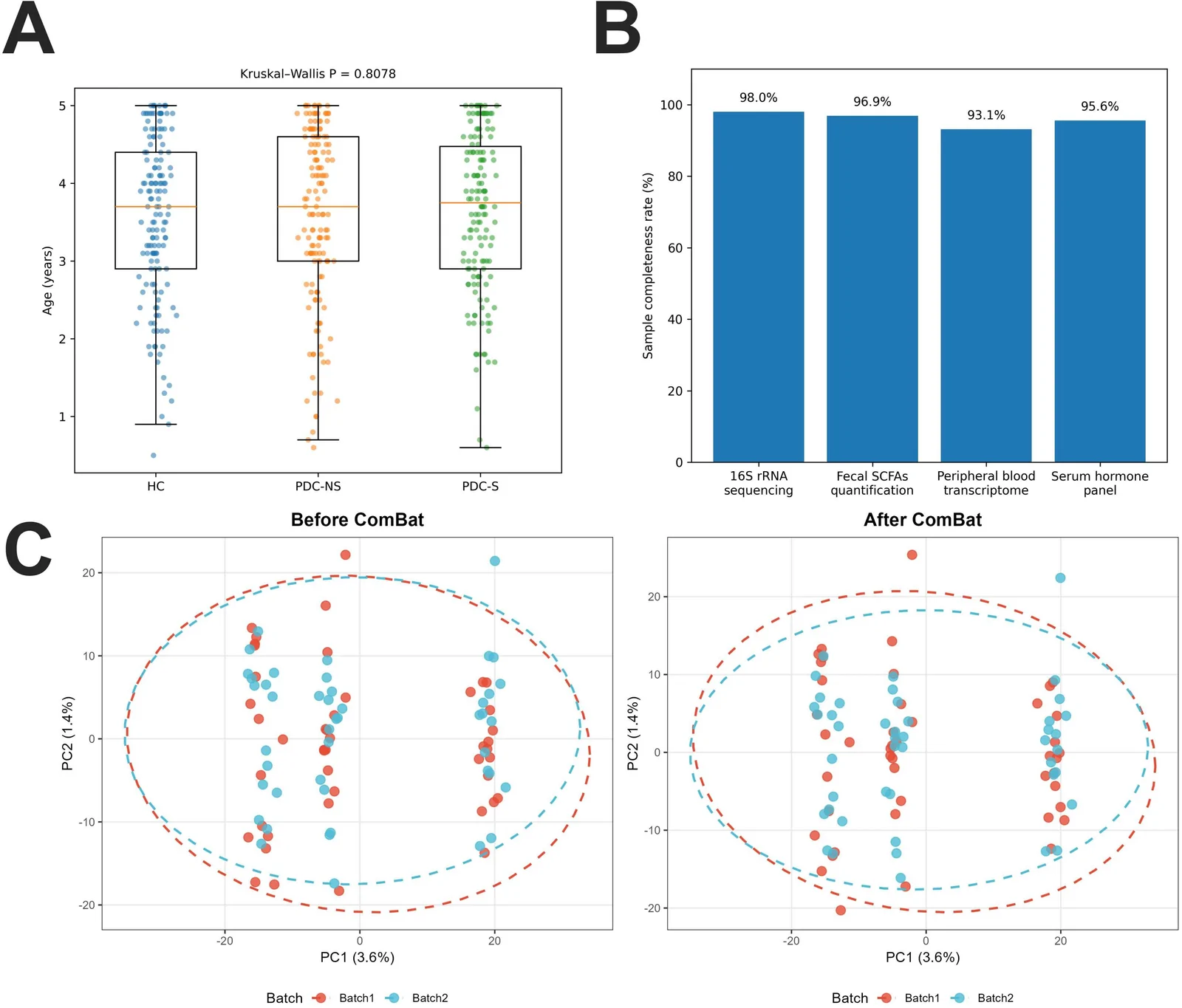
Baseline cohort characteristics, multi-omics data completeness, and batch-effect correction results. (**A**) Box-and-scatter plot showing the age distribution of children in the healthy control (HC), persistent diarrhea without short stature (PDC-NS), and persistent diarrhea with short stature (PDC-S) groups. Intergroup differences were assessed using the Kruskal–Wallis test (*P* = 0.8078). (**B**) Bar plot showing the sample completeness rates for four multi-omics assays, including 16S rRNA sequencing, fecal SCFA quantification, peripheral blood transcriptomics, and serum hormone profiling. (**C**) Principal component analysis (PCA) scatter plots showing the spatial distribution of multi-omics samples before ComBat correction (left, Before ComBat) and after ComBat correction (right, After ComBat). Red dots represent Batch 1, blue dots represent Batch 2, and dashed ellipses indicate 95% confidence intervals.

To minimize the potential influence of confounding factors on subsequent multi-omics analyses, we first evaluated the baseline characteristics of children in the HC, PDC-NS, and PDC-S groups. Analysis of age distribution showed no statistically significant differences among the three groups (Fig. 1A), indicating that the baseline characteristics were well matched and comparable across cohorts (Table 1).

**TABLE 1:** Demographic characteristics and data completeness of the three cohorts^*a*^

Characteristic	PDC-S (*n* = 150)	PDC-NS (*n* = 150)	HC (*n* = 150)	*P* value
Age (months)	24.1 (22.9–25.4)	24.4 (23.0–25.5)	24.0 (22.8–25.2)	0.81
Male, *n* (%)	78 (52.0)	77 (51.3)	77 (51.3)	0.99
16S completeness (%)	98	98	98	–
Metagenome completeness (%)	94.7	94.7	94.7	–
SCFAs completeness (%)	96.9	96.9	96.9	–
Transcriptome completeness (%)	93.1	93.1	93.1	–
Hormone panel completeness (%)	95.6	95.6	95.6	–

^*a*^ Data are presented as median (interquartile range, IQR) or frequency (percentage, %). *P* values were calculated using the Kruskal–Wallis test or chi-square test (*χ*^2^ test) as appropriate. The integrity of multi-omics data refers to the proportion of successfully processed samples relative to the total number of samples in each assay. –, not applicable; no intergroup statistical comparison was performed for data completeness indicators.

For multi-omics data quality control, we comprehensively assessed the completeness of each data set. The sample completeness rates for 16S rRNA sequencing, fecal SCFA quantification, peripheral blood transcriptomics, and serum hormone panel detection were 98.0%, 96.9%, 93.1%, and 95.6%, respectively (Fig. 1B). These results indicate that the multi-omics data sets generated in this study had high completeness and reliability, providing a robust basis for subsequent integrative analyses.

Because samples were processed in different batches, which may introduce non-biological variation or systematic bias, the ComBat algorithm was applied for batch-effect correction. PCA showed that, before ComBat correction, samples from Batch 1 and Batch 2 exhibited partial clustering and separation in the principal component space. After ComBat correction, samples from the two batches were evenly mixed, and the original batch-associated distributional differences were markedly reduced (Fig. 1C).

Together, these results demonstrate that the enrolled cohorts were well balanced in terms of baseline characteristics, that the multi-omics data sets showed high completeness, and that systematic bias caused by batch effects was successfully minimized. These quality-control procedures established a high-quality data foundation for accurately identifying biological features and regulatory axes associated with persistent diarrhea and short stature.

### Impact of persistent diarrhea on linear growth and characterization of the short-stature phenotype in children

**Fig 2:**
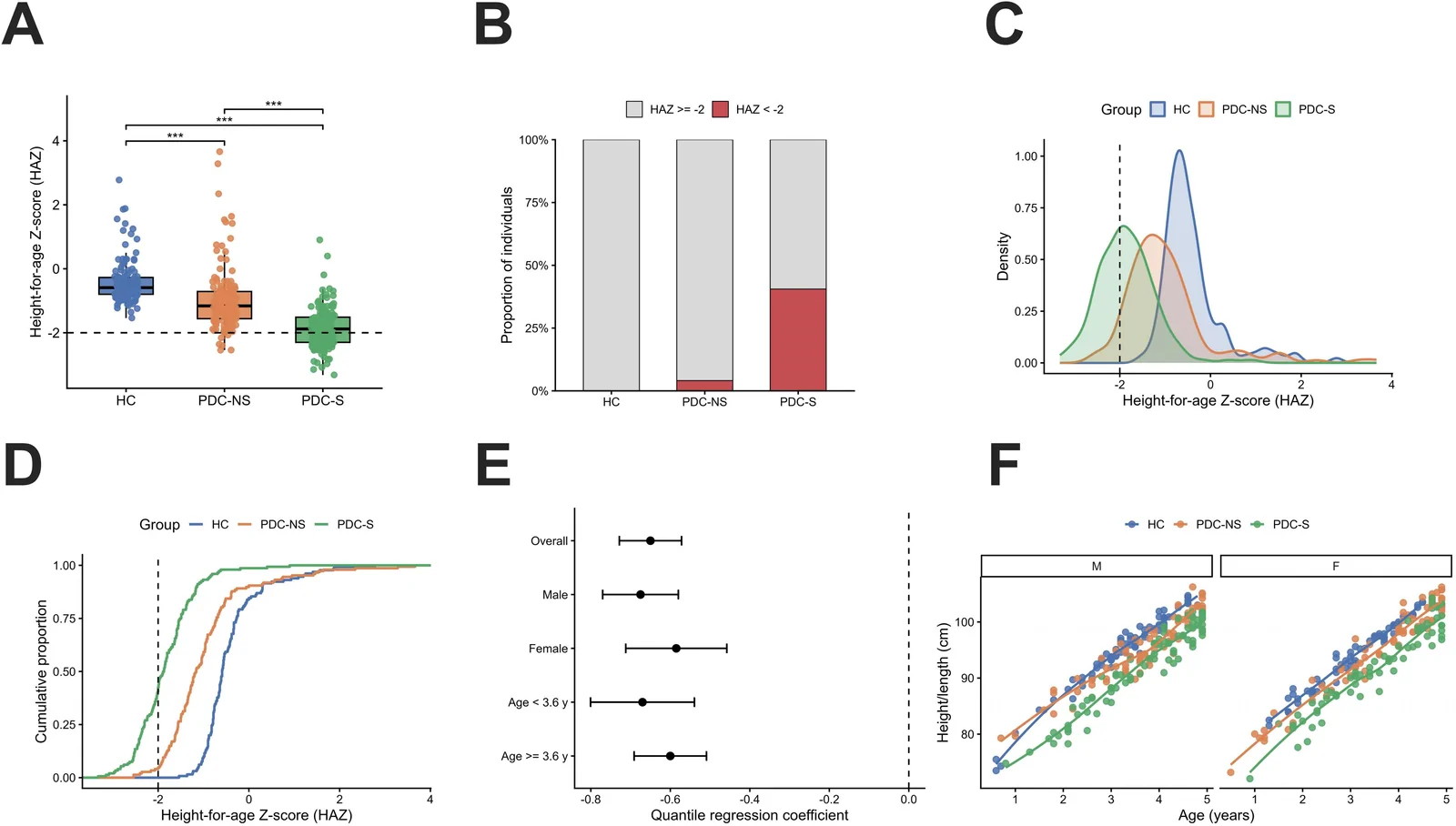
Reduced HAZ and characterization of the short-stature phenotype in children with persistent diarrhea. (**A**) Box-and-scatter plot comparing height-for-age *Z*-scores (HAZ) among the healthy control (HC), persistent diarrhea without short stature (PDC-NS), and persistent diarrhea with short stature (PDC-S) groups. The dashed line indicates the clinical threshold for short stature (HAZ = −2). ***P < 0.001. (**B**) Stacked bar plot showing the proportions of children with normal height (HAZ ≥ −2, gray) and short stature (HAZ). (**C**) Density plots showing the population distribution of HAZ scores in the three groups. (**D**) Empirical cumulative distribution function (ECDF) curves showing the cumulative distribution of HAZ scores across groups. (**E**) Forest plot showing quantile regression results assessing the negative effect of clinical group status on height in the overall population and in sex-stratified (Male/Female) and age-stratified (<3.6 years/≥3.6 years) subgroups, presented as regression coefficients. (**F**) Sex-faceted age–height scatter plots with fitted growth curves, showing the height trajectories across age in children from different groups (M, male; F, female).

To clarify the impact of persistent diarrhea on child growth and development, we comprehensively evaluated HAZ across the three groups. Boxplot analysis showed that, compared with HC, children with PDC-NS had significantly lower HAZ values. HAZ values were further reduced in children with PDC-S, with the median approaching or falling below the clinical threshold of −2 for short stature (Fig. 2A).

Proportional distribution analysis further confirmed this trend. Compared with the HC and PDC-NS groups, the proportion of individuals with HAZ < −2, corresponding to growth retardation/short stature, was markedly increased in the PDC-S group, reaching approximately 40% (Fig. 2B). Density distribution curves showed a clear leftward shift in the overall HAZ distribution of the PDC-S group, further highlighting the growth-restricted status of these children (Fig. 2C). Empirical cumulative distribution function (ECDF) analysis also showed that, at the same HAZ threshold, the PDC-S group had the highest cumulative proportion, confirming overall delayed linear growth in this group (Fig. 2D).

To exclude the potential confounding effects of sex and age, quantile regression analysis was performed. The forest plot showed that persistent diarrhea and its related phenotypes were consistently and significantly negatively associated with height in the overall population, as well as in sex-stratified subgroups (boys and girls) and age-stratified subgroups (<3.6 years and ≥3.6 years), with all regression coefficients below 0 (Fig. 2E). In addition, sex-stratified age-height fitted curves visually demonstrated that, in both boys and girls, the height trajectory of the PDC-S group remained at the lowest level across age, followed by the PDC-NS group, whereas the HC group showed the highest trajectory (Fig. 2F).

Together, these clinical analyses demonstrate that persistent diarrhea is closely associated with impaired linear growth in children. The PDC-S group exhibited a pronounced growth-retardation phenotype, and this adverse effect was independent of sex and age.

### Reduced gut microbial α-diversity in children with persistent diarrhea and short stature

**Fig 3:**
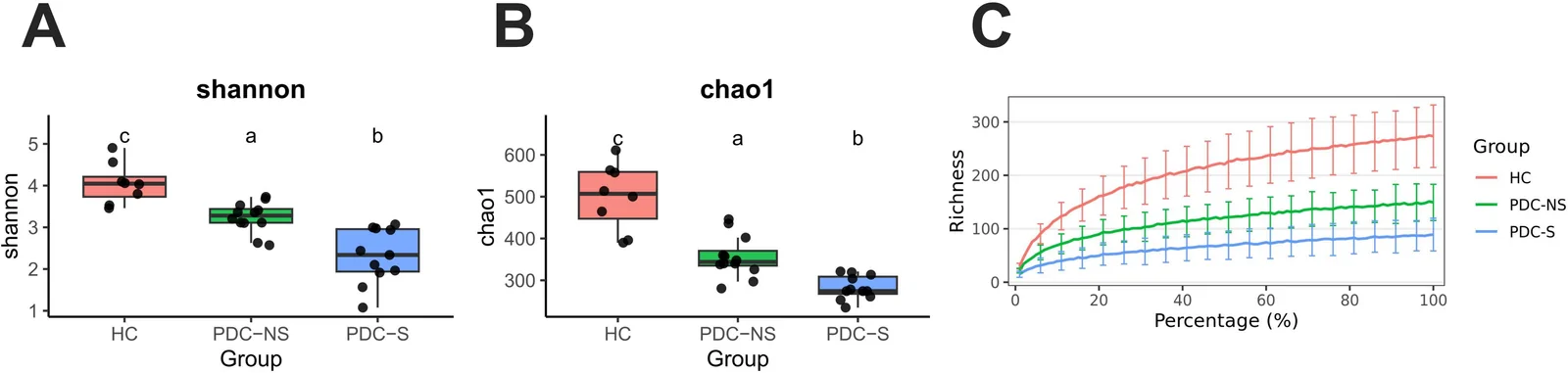
Changes in gut microbial α-diversity among the three groups of children. (**A**) Box-and-scatter plot showing the distribution of the Shannon index of gut microbiota in the healthy control (HC), persistent diarrhea without short stature (PDC-NS), and persistent diarrhea with short stature (PDC-S) groups, used to evaluate community diversity. (**B**) Box-and-scatter plot showing the distribution of the Chao1 index among the three groups, used to evaluate species richness. Different lowercase letters above the box plots (**A–C**) indicate statistically significant differences among groups, such as *p* < 0.05. (**C**) Rarefaction curves showing changes in observed species richness across different sequencing-depth proportions in each group. Error bars indicate standard deviation. Curves approaching a plateau indicate sufficient sequencing depth.

To evaluate the effects of persistent diarrhea and its accompanying short-stature phenotype on the overall structure of the pediatric gut microecosystem, we analyzed gut microbial α-diversity across the three groups based on 16S rRNA sequencing data. The Shannon index was used to assess community diversity. Compared with the HC group, the PDC-NS group showed a significant decrease in the Shannon index, whereas the PDC-S group exhibited a further significant reduction (Fig. 3A). Similarly, the Chao1 index, which reflects community richness, showed a consistent trend, with the highest value in the HC group, an intermediate value in the PDC-NS group, and the lowest value in the PDC-S group; the differences among groups were statistically significant (Fig. 3B).

In addition, rarefaction curves were generated to evaluate species richness across samples. As sequencing depth increased, the curves of all three groups gradually approached a plateau, indicating that the sequencing depth was sufficient to capture most microbial species in the samples and that the data set was reliable (Fig. 3C). The rarefaction curves also visually reflected differences in species richness across different sequencing depths: the HC group showed the highest richness, followed by the PDC-NS group, whereas the PDC-S group consistently displayed the lowest species richness.

Together, these results indicate that persistent diarrhea markedly disrupts gut microbial homeostasis in children, leading to reduced microbial diversity and richness. This microecological disturbance was more pronounced in children with persistent diarrhea and short stature.

### Marked shift in gut microbial community structure (β-diversity) in children with persistent diarrhea and short stature

**Fig 4:**
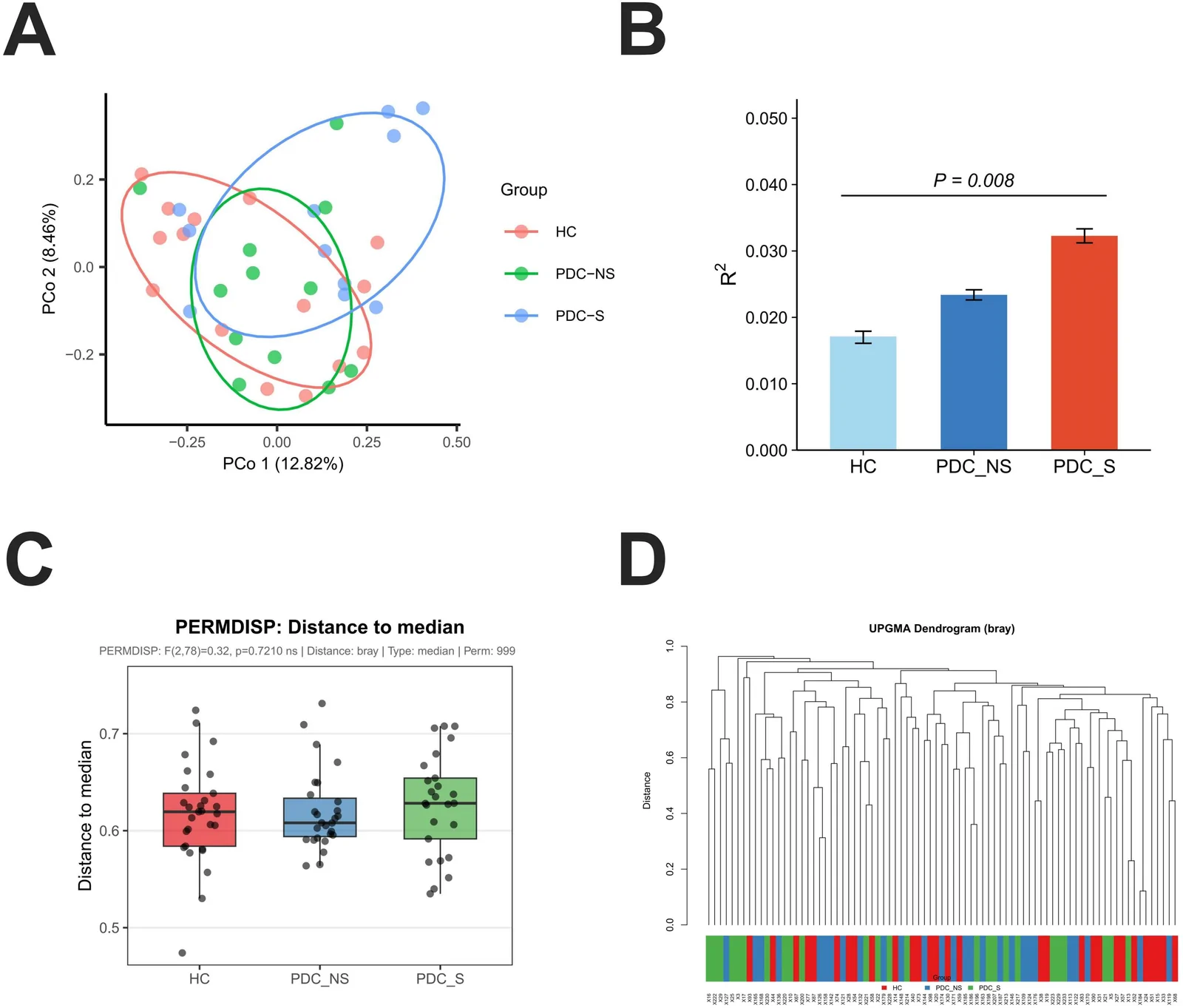
Separation of gut microbial community structure based on β-diversity analysis. (**A**) Principal coordinate analysis (PCoA) scatter plot showing differences in the spatial distribution of gut microbial community structure among the three groups (HC, PDC-NS, and PDC-S). Outer ellipses indicate 95% confidence intervals. (**B**) Bar plot showing the statistical significance of community-structure differences assessed by multivariate statistical testing (*P* = 0.008). Bars represent the explained variation or test statistic, and error bars indicate standard deviation. (**C**) PERMDISP box-and-scatter plot showing the distance of samples to the group median among the three groups, used to assess homogeneity of community dispersion. *P* = 0.7210 indicates no significant difference in within-group dispersion (ns). (**D**) UPGMA hierarchical clustering dendrogram based on Bray–Curtis distances, showing similarity relationships in microbial community structure among samples.The color legend at the bottom indicates sample group information: red, HC; green, PDC-NS; and blue, PDC-S.

After confirming the reduction in α-diversity, we further evaluated differences in overall gut microbial community structure among the three groups using β-diversity analysis. PCoA based on the distance matrix showed that samples from the HC, PDC-NS, and PDC-S groups partially overlapped in the ordination space, but their core distribution regions showed clear shifts and separation trends (Fig. 4A).

To quantify these structural differences in community composition, statistical testing was performed. The results showed that the overall gut microbial community structure differed significantly among the three groups (Fig. 4B). To further assess within-group variation, PERMDISP analysis (distance to median) was conducted. No significant differences in within-group dispersion were observed, indicating that the structural differences identified among groups were attributable to genuine biological changes in community composition rather than unequal within-group variance (Fig. 4C).

In addition, an unweighted pair-group method with arithmetic mean (UPGMA) hierarchical clustering dendrogram based on the Bray–Curtis distance matrix further reflected similarities in community composition among samples. The color strips at the bottom showed that, although samples from the three groups were not completely physically segregated, many PDC-S and PDC-NS samples tended to cluster in specific branches, exhibiting group-specific aggregation patterns distinct from those of the HC group (Fig. 4D).

Together, these β-diversity results indicate that persistent diarrhea and its accompanying short-stature phenotype not only reduce gut microbial diversity, but also substantially reshape the overall architecture of the gut microbial community.

### Marked depletion of core butyrate-producing bacteria in children with persistent diarrhea and short stature

**Fig 5:**
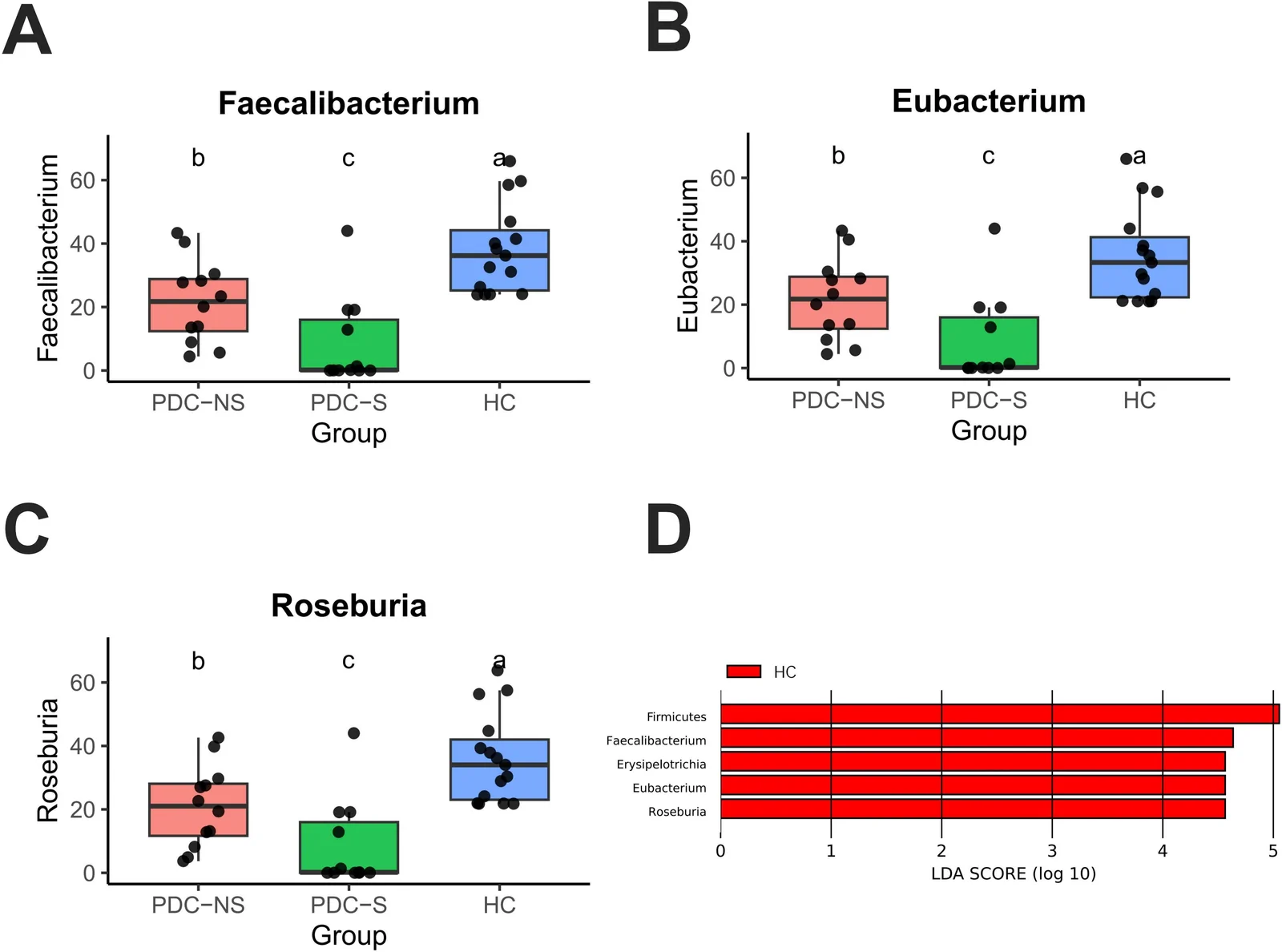
Significant changes in the abundance of butyrate-producing bacteria among children with different growth phenotypes. (**A–C**) Box-and-scatter plots showing the distributions of the relative abundances of *Faecalibacterium* (**A**), *Eubacterium* (**B**), and *Roseburia* (**C**) in the gut microbiota of children from the persistent diarrhea without short stature (PDC-NS), persistent diarrhea with short stature (PDC-S), and healthy control (HC) groups. Different lowercase letters above the box plots (**A–C**) indicate statistically significant pairwise differences among groups, such as *P* < 0.05. (**D**) LEfSe analysis presented as an LDA score bar plot, showing bacterial taxonomic biomarkers significantly enriched in the healthy control (HC) group, using the screening criterion of LDA score (log10) > 4.0.

To further investigate the specific taxa underlying gut microbial dysbiosis, we first used linear discriminant analysis effect size (LEfSe) to identify differentially abundant microbial biomarkers. Notably, *Faecalibacterium*, *Eubacterium*, and *Roseburia* are well-recognized core butyrate-producing genera in the gut. We therefore further quantified the relative abundances of these three beneficial genera across the three groups of children. Boxplot analysis showed that, compared with the HC group, children in the PDC-NS group exhibited significantly reduced abundances of *Faecalibacterium* (Fig. 5A), *Eubacterium* (Fig. 5B), and *Roseburia* (Fig. 5C). In the PDC-S group, the relative abundances of these three butyrate-producing genera were further markedly decreased, approaching near depletion.

LEfSe analysis further showed that multiple specific bacterial taxa were significantly enriched in the HC group. In particular, the phylum Firmicutes and its affiliated taxa, including the genera *Faecalibacterium*, *Eubacterium*, and *Roseburia*, as well as the class Erysipelotrichia, all showed log LDA scores greater than 4, indicating that they represent important signature taxa associated with a healthy gut microecological state (Fig. 5D).

Together, these findings suggest that persistent diarrhea is accompanied by a substantial loss of core beneficial butyrate-producing bacteria in the gut. Moreover, the degree of depletion of these butyrate-producing taxa appears to be closely associated with growth restriction, as reflected by the short-stature phenotype. These results provide an important taxonomic basis for subsequent investigation of the role of SCFAs in growth-regulatory pathways.

### PICRUSt2 functional prediction reveals markedly impaired SCFA biosynthesis pathways in children with persistent diarrhea and short stature

Given that the preceding taxonomic analysis showed marked depletion of core butyrate-producing bacteria in children with persistent diarrhea and short stature, we further used PICRUSt2 to predict the metabolic functions of the gut microbiota based on 16S rRNA data, with a particular focus on MetaCyc pathways related to SCFA biosynthesis.

**Fig 6:**
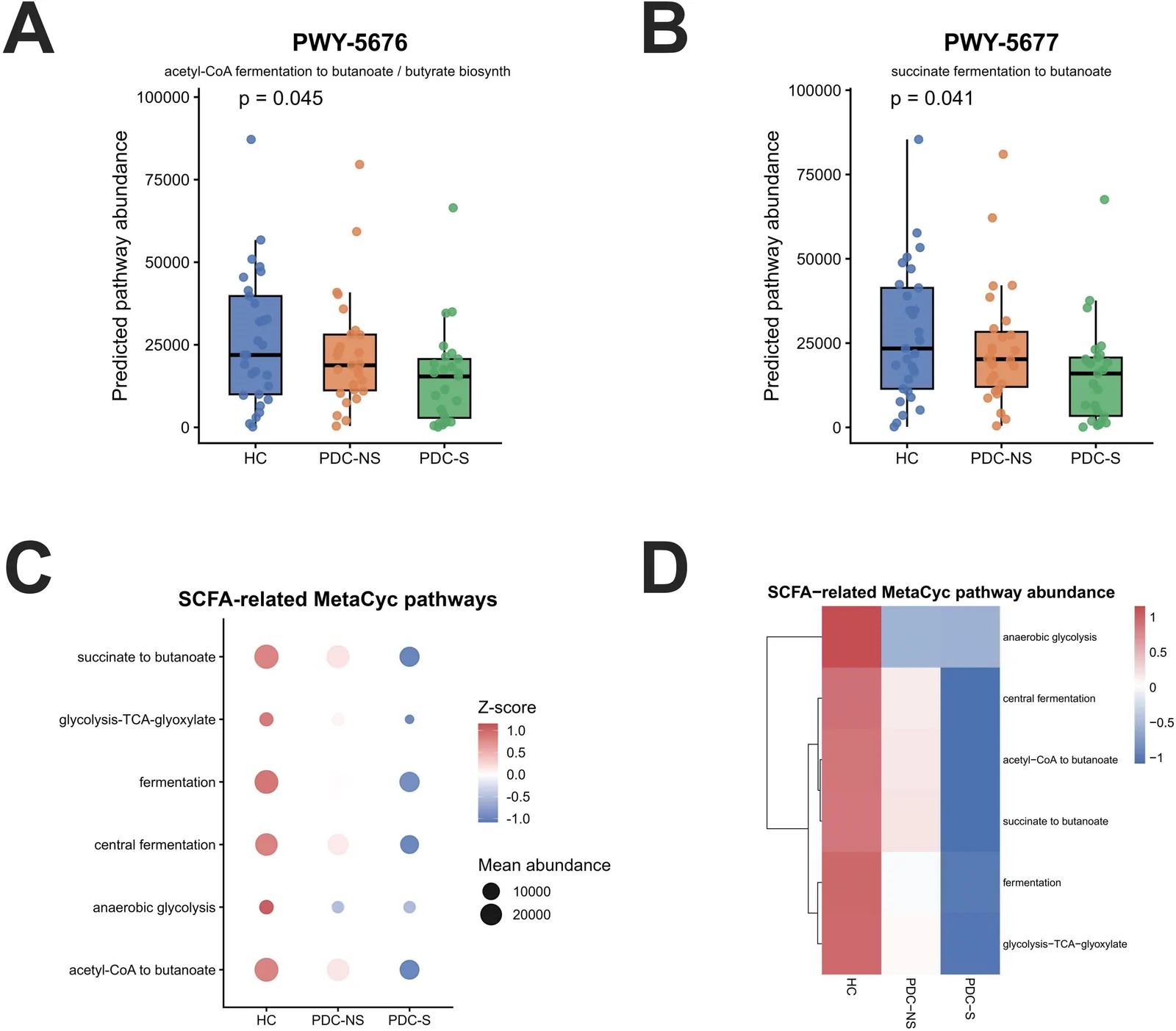
PICRUSt2 prediction reveals marked impairment of gut SCFA- and butyrate-biosynthesis pathways in children with short stature. (**A**) Box-and-scatter plot showing the predicted abundance of the MetaCyc pathway PWY-5676 (acetyl-CoA fermentation to butanoate) in the gut microbiota of the three groups of children (*P* = 0.045). (**B**) Box-and-scatter plot showing the predicted abundance of the MetaCyc pathway PWY-5677 (succinate fermentation to butanoate) in the gut microbiota of the three groups (*P* = 0.041). (**C**) Bubble plot showing changes in the abundances of multiple metabolic pathways related to short-chain fatty acid (SCFA) biosynthesis. Bubble size represents the mean predicted abundance of each pathway, and color intensity represents row-standardized abundance values (*Z*-scores; red indicates high abundance and blue indicates low abundance). (**D**) Heatmap showing the relative abundances (*Z*-scores) and clustering patterns of the above SCFA-related metabolic pathways among the healthy control (HC), persistent diarrhea without short stature (PDC-NS), and persistent diarrhea with short stature (PDC-S) groups.

Abundance analysis showed that two key butyrate biosynthesis pathways—PWY-5676 (acetyl-CoA fermentation to butanoate; Fig. 6A) and PWY-5677 (succinate fermentation to butanoate; Fig. 6B)—differed significantly among the three groups (*P* = 0.045 and *P* = 0.041, respectively). Compared with the HC group, the predicted abundances of these two pathways were reduced in the PDC-NS group and reached their lowest levels in the PDC-S group.

To more comprehensively evaluate changes in SCFA-related metabolic networks, we extracted and visualized the predicted abundances of multiple SCFA-related pathways. The bubble plot (Fig. 6C) and clustered heatmap (Fig. 6D) showed highly consistent patterns: a series of energy metabolism and SCFA biosynthesis pathways, including anaerobic glycolysis, central fermentation, and multiple substrate-to-butyrate conversion pathways (acetyl-CoA to butanoate and succinate to butanoate), exhibited relatively high abundances (red color/large bubbles) in the HC group, were downregulated in the PDC-NS group, and showed the most pronounced decline and suppression (blue color/small bubbles) in the PDC-S group.

Together, these functional predictions are highly consistent with the taxonomic loss of butyrate-producing genera and collectively demonstrate that the gut microbiota of children with persistent diarrhea and short stature is severely impaired in its capacity to produce short-chain fatty acids, particularly butyrate.

### Fecal SCFA levels are significantly reduced in children with short stature and positively correlate with linear growth indicators

Based on the preceding 16S rRNA sequencing and functional prediction results, which indicated impairment of SCFA-producing bacteria and related metabolic pathways, we further performed targeted metabolomic analysis to accurately quantify the absolute levels of major SCFAs in fecal samples from the three groups of children.

**Fig 7:**
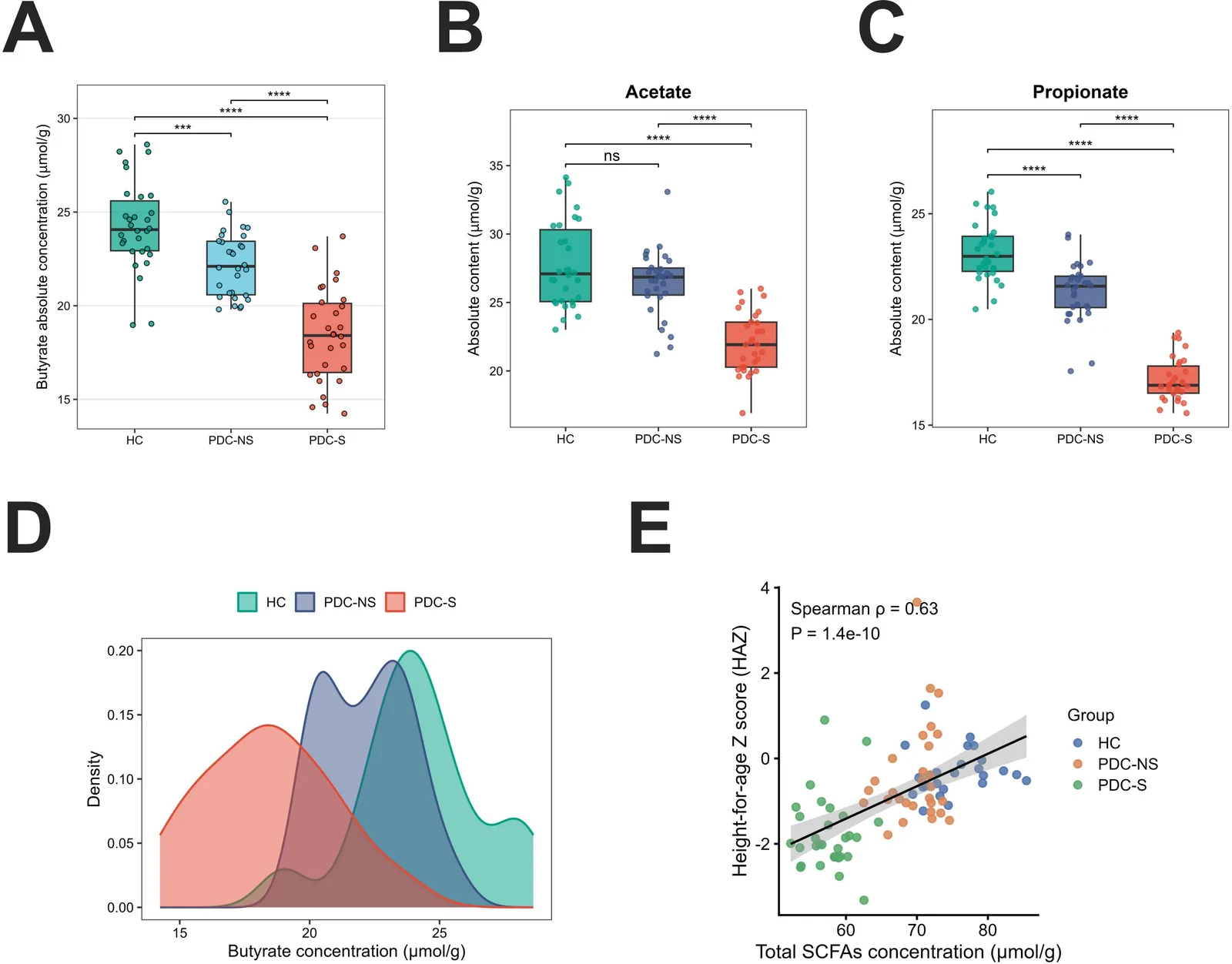
Marked reduction of fecal short-chain fatty acids (SCFAs) in children with PDC and their correlation with HAZ. (**A–C**) Box-and-scatter plots quantitatively comparing the absolute concentrations of fecal butyrate (**A**), acetate (**B**), and propionate (**C**) among the healthy control (HC), persistent diarrhea without short stature (PDC-NS), and persistent diarrhea with short stature (PDC-S) groups (unit: μmol/g). Appropriate statistical tests were used for intergroup comparisons. ns indicates no significant difference; ***P < 0.001; ****P < 0.0001. (**D**) Density plot showing the population distribution of fecal butyrate concentrations in the three groups of children, with the PDC-S group showing a clear leftward shift toward lower concentrations. (**E**) Scatter plot with fitted line showing the Spearman correlation between fecal total SCFA concentration and height-for-age *Z*-score (HAZ) in the study cohort. The black line represents the linear regression fit, and the gray shaded area indicates the 95% confidence interval (Spearman *ρ* = 0.63, *P* = 1.4e−10). Dots of different colors represent samples from different groups.

Quantitative analysis showed that all three major SCFAs were markedly deficient in children with the short-stature phenotype. Specifically, compared with the HC group, fecal butyrate and propionate concentrations were significantly reduced in the PDC-NS group, and these two metabolites showed the most pronounced decreases in the PDC-S group (Fig. 7A and C). For acetate, although no significant difference was observed between the HC and PDC-NS groups, the acetate level in the PDC-S group was still significantly lower than those in the other two groups (Fig. 7B).

The population-level distribution of butyrate, a core metabolite, was further visualized using density plots. The butyrate concentration curve in the PDC-S group was clearly shifted toward the low-concentration range, confirming severe depletion of the intestinal butyrate pool in these children (Fig. 7D).

To clarify the relationship between intestinal SCFA levels and clinical growth status, we performed Spearman correlation analysis between fecal total SCFA concentrations and HAZ, an indicator of linear growth. The results showed a highly significant positive correlation between total intestinal SCFA levels and HAZ (Fig. 7E). This finding strongly suggests that long-term deficiency of gut microbiota-derived SCFAs, particularly butyrate, may represent a key metabolic driver of growth retardation and short stature in children with persistent diarrhea.

### Peripheral blood transcriptomics reveals pronounced Th17/Treg imbalance and activation of inflammatory pathways in children with short stature

**Fig 8:**
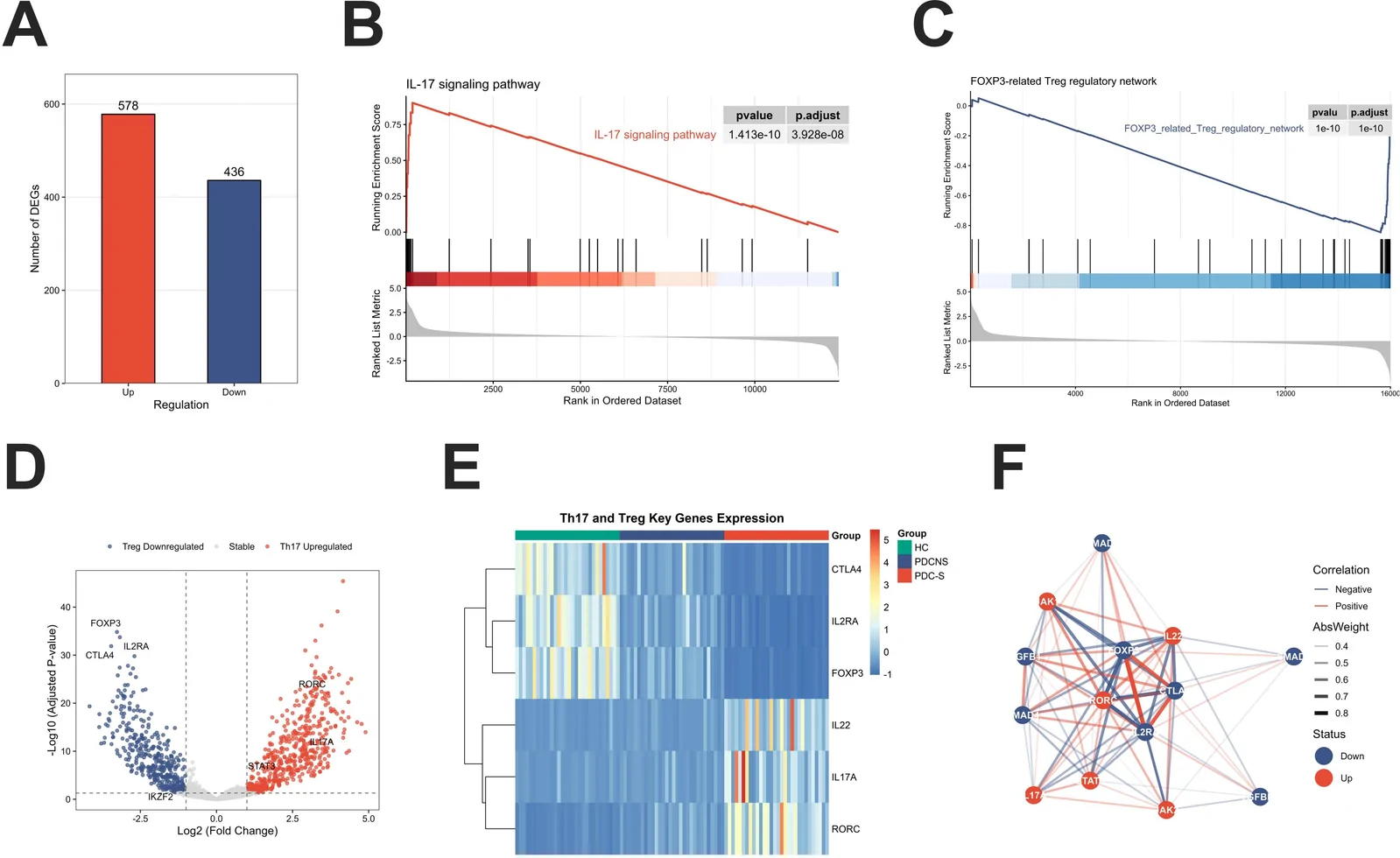
Peripheral blood transcriptomic analysis reveals Th17/Treg imbalance and abnormal immune-inflammatory signaling in affected children. (**A**) Bar plot showing the numbers of differentially expressed genes (DEGs), with red indicating significantly upregulated genes (578 genes) and blue indicating significantly downregulated genes (436 genes). (**B and C**) Gene set enrichment analysis (GSEA) plots showing significant positive enrichment of the IL-17 signaling pathway (**B**) and significant negative enrichment of the FOXP3-related Treg regulatory network (**C**). (**D**) Volcano plot showing the distribution of DEGs, with key significantly upregulated Th17-related genes highlighted in red and significantly downregulated Treg-related genes highlighted in blue. (**E**) Clustered heatmap showing differences in the relative expression levels of key Th17 and Treg marker genes among the healthy control (HC), persistent diarrhea without short stature (PDC-NS), and persistent diarrhea with short stature (PDC-S) groups. Colors range from blue to red, indicating low- to high-expression levels. (**F**) Co-expression regulatory network of core differential immune genes. Node color indicates gene upregulation (red) or downregulation (blue), edge color indicates positive correlation (red) or negative correlation (blue), and edge thickness represents the absolute value of correlation weight.

To investigate systemic immune responses associated with gut microecological dysbiosis, we performed an in-depth analysis of peripheral blood transcriptomic data. Differential expression analysis identified 578 significantly upregulated and 436 significantly downregulated DEGs in children with the short-stature phenotype (Fig. 8A).

To clarify the biological functions mediated by these DEGs, GSEA was performed. The results showed marked positive enrichment and activation of the IL-17 signaling pathway (Fig. 8B). In contrast, the FOXP3-related Treg regulatory network was significantly negatively enriched and suppressed (Fig. 8C). These pathway-level alterations strongly suggest that the peripheral immune system of affected children is shifted toward a pro-inflammatory state.

We further validated this imbalance at the gene level. The DEG volcano plot (Fig. 8D) showed that core Th17-related pro-inflammatory transcription factors, such as *RORC* and *STAT3*, and effector molecules, such as *IL17A*, were significantly upregulated. In contrast, Treg signature genes with immunosuppressive functions, including *FOXP3*, *IL2RA*, *CTLA4*, and *IKZF2*, were significantly downregulated. The clustered heatmap (Fig. 8E) further confirmed that the expression levels of Th17 marker genes, including *IL22*, *IL17A*, and *RORC*, were markedly higher in the PDC-S and PDC-NS groups than in the HC group, whereas Treg marker genes, including *CTLA4*, *IL2RA*, and *FOXP3*, were sharply reduced.

Finally, the gene co-expression network (Fig. 8F) revealed complex regulatory relationships among these core immune genes. Upregulated Th17 module genes, shown as red nodes, exhibited extensive strong negative correlations with downregulated Treg module genes, shown as blue nodes and connected by blue edges, highlighting the antagonistic interaction between these two T-cell subsets during disease progression.

Together, the peripheral blood transcriptomic data demonstrate a marked disruption of Th17/Treg immune balance in children with persistent diarrhea and short stature. This sustained systemic inflammatory state may represent a key immune-mediated mechanism linking intestinal metabolite deficiency to impaired systemic growth and development.

### Clinical and omics-integrated analyses reveal marked suppression of the GH/IGF-1 growth axis in children with short stature

To determine how gut microecological dysbiosis and systemic inflammation ultimately lead to restricted somatic growth, we performed integrated clinical and transcriptomic analyses of key indicators of the GH/IGF-1 axis, the central endocrine pathway regulating linear growth in children.

**Fig 9:**
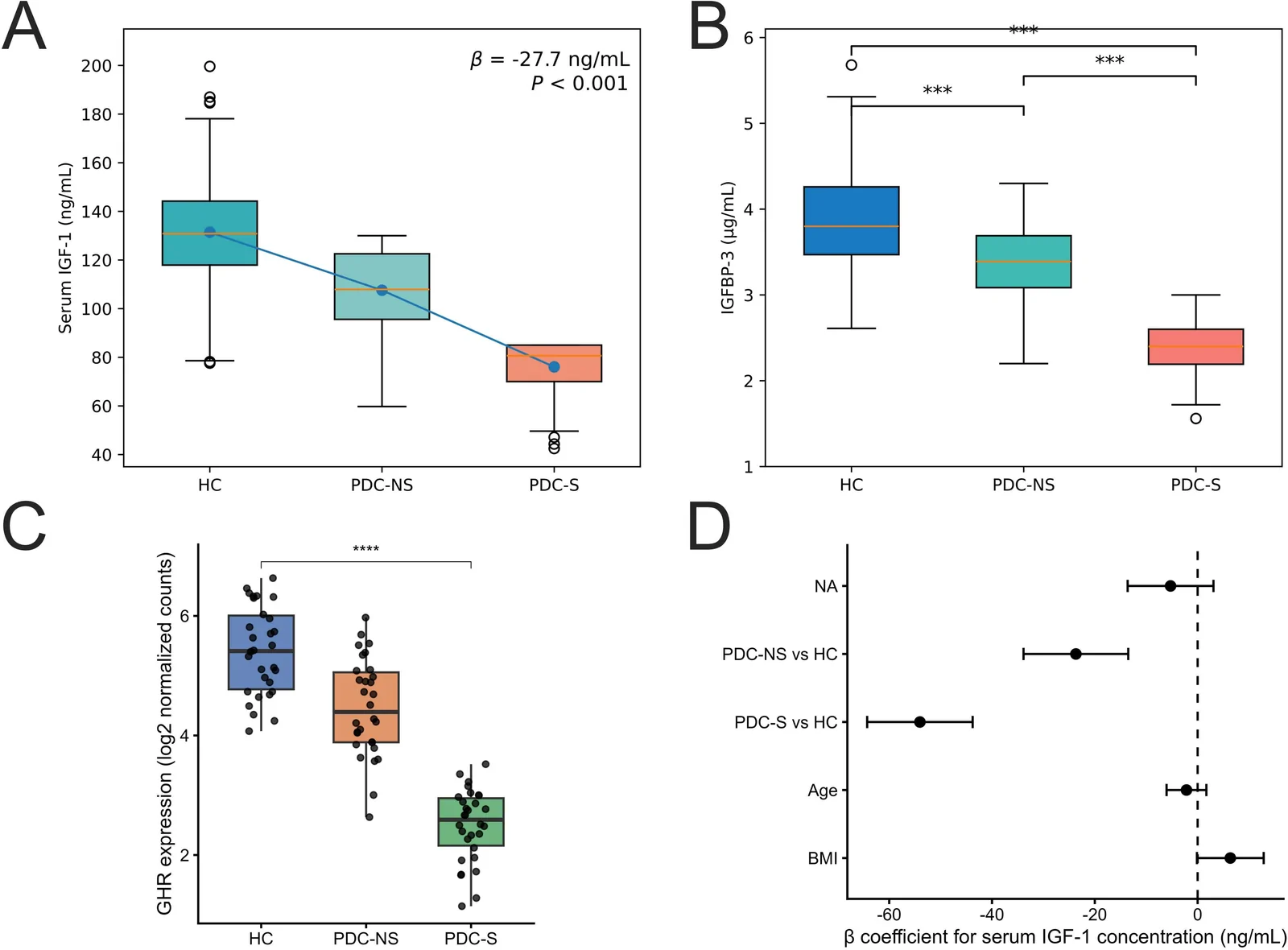
Marked reduction of key indicators of the GH/IGF-1 growth-regulatory axis in children with persistent diarrhea and short stature. (**A**) Box plot with trend line showing the distribution and decreasing trend of serum IGF-1 concentrations among the healthy control (HC), persistent diarrhea without short stature (PDC-NS), and persistent diarrhea with short stature (PDC-S) groups. (**B**) Box plot showing significant differences in serum IGFBP-3 concentrations among the three groups. (**C**) Box-and-scatter plot showing changes in growth hormone receptor (*GHR*) gene expression levels among the three groups based on peripheral blood transcriptomic data. (**D**) Forest plot showing the results of multivariable linear regression analysis, evaluating the independent effects of clinical group status (PDC-NS vs HC and PDC-S vs HC) and covariates, including age and BMI, on serum IGF-1 concentrations.

Serological analysis showed that, compared with the HC group, serum IGF-1 levels were reduced in children with PDC-NS, whereas children with PDC-S exhibited the most pronounced decrease in IGF-1 levels (Fig. 9A). Similarly, serum levels of IGFBP-3, the major circulating binding protein of IGF-1, were also markedly reduced in the PDC-S group (Fig. 9B), suggesting a severe deficiency in the biologically available IGF-1 reserve.

Moreover, peripheral blood transcriptomic data further revealed abnormalities at the receptor level. The expression of *GHR*, a key receptor gene mediating GH signaling, was significantly lower in the PDC-S group than in the HC and PDC-NS groups (Fig. 9C), indicating that GH target responsiveness was also substantially impaired.

To exclude the potential influence of confounding factors such as age and body mass index (BMI), multivariable regression analysis was performed. The forest plot confirmed that disease-group status, particularly membership in the PDC-S group, was an independent and the strongest negative driver of reduced serum IGF-1 levels (Fig. 9D).

Together, these results demonstrate that children with persistent diarrhea and short stature exhibit profound suppression of the GH/IGF-1 growth axis at both the ligand level (IGF-1/IGFBP-3) and receptor level (*GHR*). This impairment of endocrine regulation is likely to represent a key terminal mechanism linking microbiota–metabolism–immune dysregulation to the final short-stature phenotype.

### Cross-omics correlation analysis reveals a close link between gut butyrate and the immune–growth endocrine axis

To elucidate the coordinated role of the gut microecology–immune–endocrine regulatory axis from a multi-dimensional perspective, we performed cross-omics correlation analysis integrating fecal butyrate concentrations detected by metabolomics with immune transcriptomic features, including *FOXP3* and *IL17A* expression, and clinical serological indicators, particularly IGF-1.

**Fig 10:**
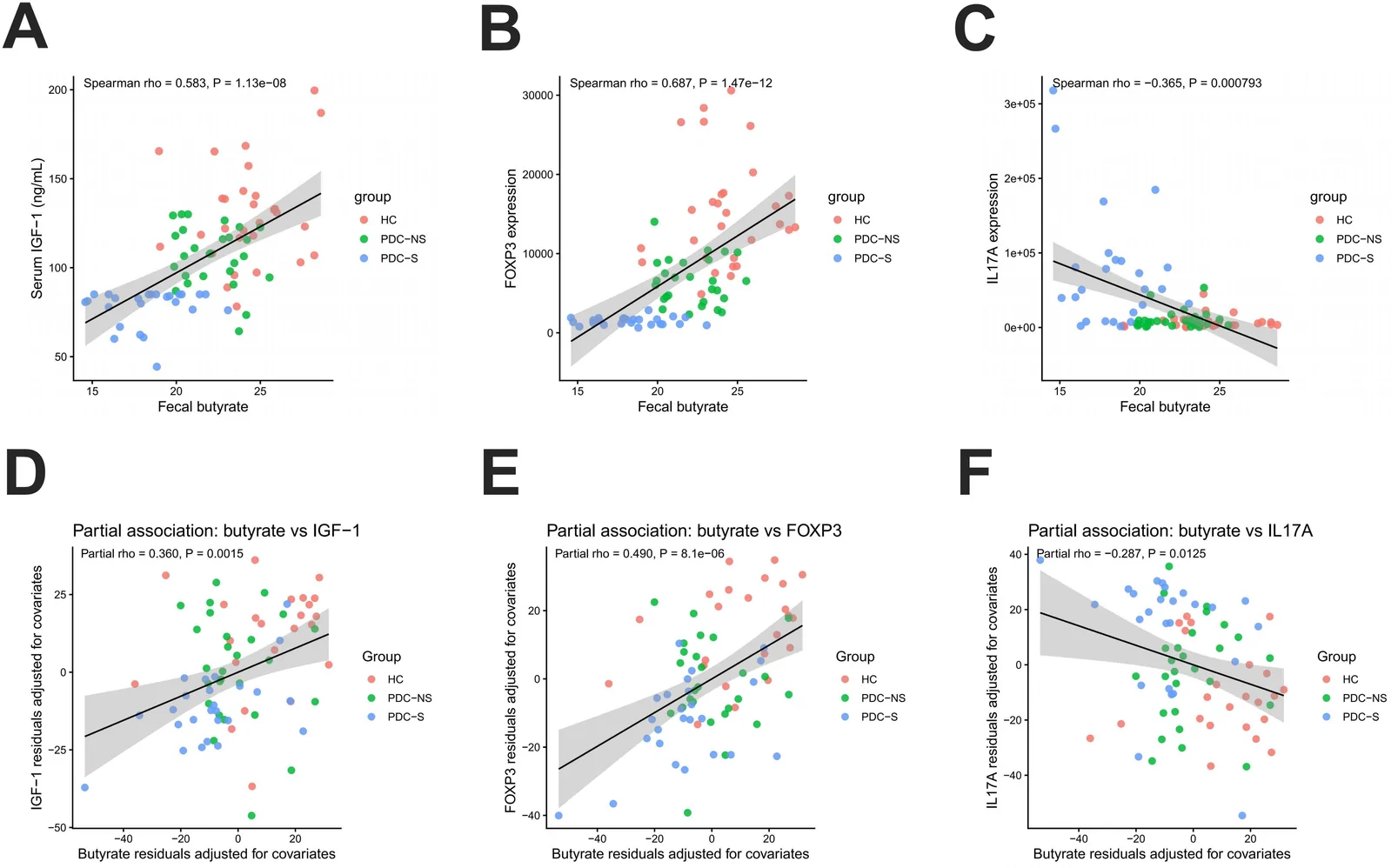
Cross-omics analysis reveals significant correlations between fecal butyrate levels and immune and growth indicators. (**A–C**) Scatter plots showing unadjusted Spearman correlation analyses based on raw clinical and omics data: correlations of fecal butyrate concentration with serum IGF-1 concentration (**A**), peripheral blood *FOXP3* gene expression (**B**), and *IL17A* gene expression (**C**). (**D–F**) Scatter plots showing partial association analyses after adjustment for covariates such as age and BMI. The *x*- and *y*-axes represent residual values after model adjustment. The black line indicates the linear regression fit, and the gray shaded area represents the 95% confidence interval. Dot colors indicate different groups: red, healthy control (HC); green, persistent diarrhea without short stature (PDC-NS); and blue, persistent diarrhea with short stature (PDC-S).

Spearman correlation scatter plots showed a highly significant positive correlation between fecal butyrate levels and serum IGF-1 concentrations, which reflect somatic growth status (Fig. 10A). Regarding immune indicators, butyrate concentration was strongly positively correlated with the expression of *FOXP3*, the core transcription factor of Treg cells (Fig. 10B), but significantly negatively correlated with the expression of *IL17A*, a key effector molecule of Th17 cells (Fig. 10C). These results suggest that butyrate deficiency is accompanied by intensified pro-inflammatory immune activation and reduced growth factor levels.

To exclude the potential influence of confounding covariates, including age, sex, and BMI, we further performed partial association analysis. After covariate adjustment, residual-based scatter plots confirmed that butyrate remained independently and positively associated with IGF-1 (Fig. 10D) and *FOXP3* (Fig. 10E). Meanwhile, the independent negative association between butyrate and *IL17A* was also preserved (Fig. 10F).

Together, these cross-omics analyses strongly demonstrate that reduced gut butyrate levels are closely associated with peripheral Th17/Treg immune imbalance, inflammatory activation, and systemic suppression of IGF-1 synthesis. These findings delineate a clear metabolite-mediated butyrate–immune–growth regulatory cascade.

### XGBoost-based machine learning identifies key microbial features driving gut butyrate metabolism

To determine which specific gut microbial taxa and functional pathways drive changes in butyrate levels in affected children, we constructed regression models to predict fecal butyrate concentrations based on 16S rRNA-derived omics features, including species abundance and PICRUSt2-predicted pathway abundance.

**Fig 11:**
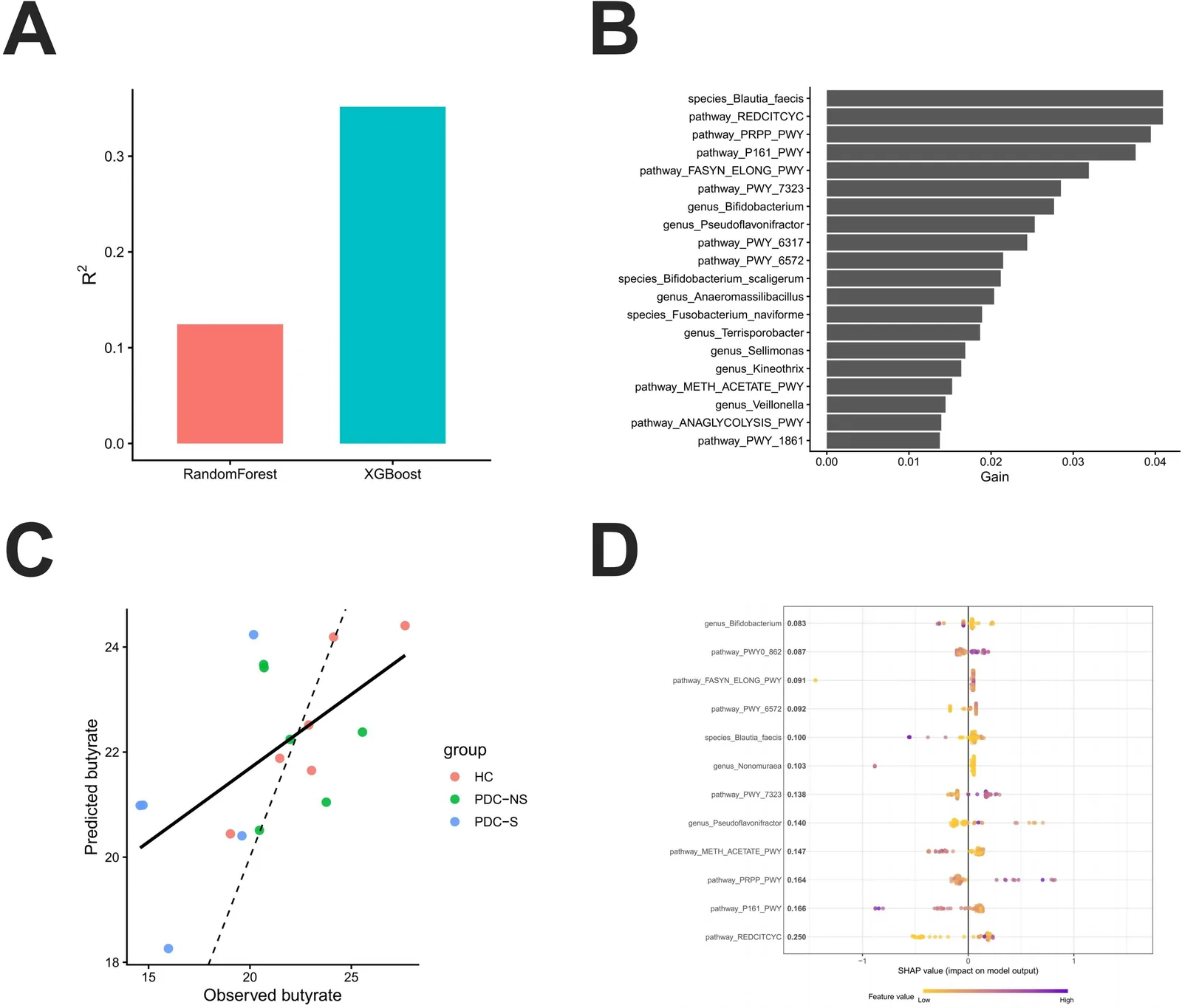
Machine learning models identify key microbial features driving butyrate production. (**A**) Bar plot comparing the performance of random forest and XGBoost regression models in predicting fecal butyrate levels, as assessed by the coefficient of determination (*R*^2^). XGBoost showed superior performance. (**B**) Bar plot showing the top-ranked microbial taxa and metabolic pathway features according to Gain-based importance scores in the XGBoost model. (**C**) Scatter plot showing the correlation between XGBoost-predicted butyrate concentrations and observed butyrate concentrations. The solid line represents the linear fitted line, and dot colors indicate different participant groups. (**D**) SHAP summary plot explaining model output. The *y*-axis shows feature names, and the *x*-axis shows SHAP values, representing the impact of each feature on the prediction. Each dot represents one sample, and dot color indicates the relative abundance of the corresponding feature, ranging from yellow to purple to indicate low to high abundance. Positive SHAP values indicate increased predicted butyrate levels, whereas negative SHAP values indicate decreased predicted values.

We first compared two commonly used tree-based ensemble learning algorithms: random forest and extreme gradient boosting (XGBoost). Model performance evaluation showed that the XGBoost model achieved a significantly higher goodness of fit (*R*^2^) than the random forest model in the current data set (Fig. 11A).

Based on the better-performing XGBoost model, we extracted and ranked the features with the greatest contribution to butyrate prediction according to gain-based feature importance. The results showed that key species such as *Blautia faecis*, a known butyrate-producing beneficial bacterium, and *Bifidobacterium*, together with several core energy and material metabolism pathways, including pathway_REDCITCYC, pathway_PRPP_PWY, and pathway_P161_PWY, were the most important drivers determining intestinal butyrate levels (Fig. 11B). Scatter fitting between the observed butyrate concentrations and XGBoost-predicted values showed a clear positive correlation, confirming the reliable predictive performance of the model (Fig. 11C).

To further clarify how these features influenced butyrate production, we applied the SHAP (SHapley Additive exPlanations) attribution framework. The SHAP summary plot visually revealed the relationship between feature values and the direction of butyrate prediction. For example, higher abundances of metabolic pathways such as pathway_REDCITCYC and pathway_P161_PWY, as well as the species *Blautia faecis*, were associated with positive SHAP values, indicating that higher levels of these microbial features directly promoted butyrate accumulation. Conversely, low feature abundance was closely associated with reduced butyrate concentrations (Fig. 11D).

Together, by applying an interpretable machine learning model, we not only achieved cross-omics prediction of butyrate levels using microbiome-derived features, but also precisely identified the microbial nodes and metabolic pathway defects responsible for butyrate depletion in children with persistent diarrhea. These findings provide precise targets for subsequent microbiota-directed interventions.

### Multi-omics feature-integrated machine learning model accurately predicts the risk of short stature in children

To translate the previously identified butyrate–immune–IGF-1 regulatory axis into a clinically applicable tool, we constructed machine learning classification models to predict the risk of short stature in children with persistent diarrhea. An Elastic Net algorithm was used, and different layers of omics features were gradually incorporated to compare model performance.

**Fig 12:**
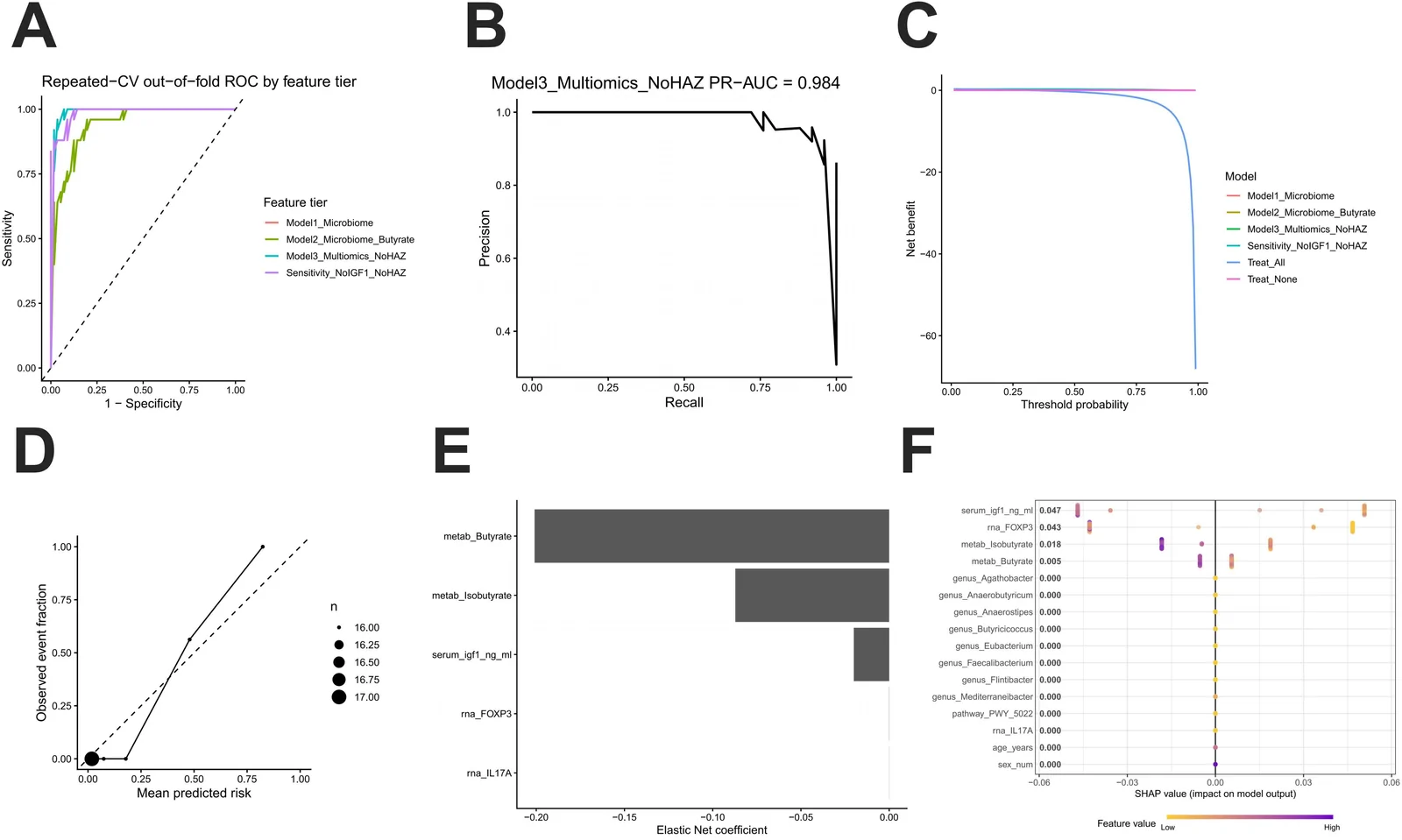
Machine learning model based on multi-omics features accurately predicts the risk of short-stature phenotype in affected children. (**A**) Receiver operating characteristic (ROC) curves comparing the repeated cross-validation performance of models constructed using different feature combinations, including microbiome-only, microbiome plus butyrate, and integrated multi-omics models. (**B**) Precision–recall (PR) curve and PR-AUC value of the optimal integrated multi-omics model (Model3_Multiomics). (**C**) Decision curve analysis (DCA) evaluating the clinical net benefit of each model across different threshold probabilities. (**D**) Calibration curve showing the agreement between the short-stature risk predicted by the multi-omics model and the observed incidence. The dashed line represents ideal perfect calibration. (**E**) Bar plot showing the key non-zero features retained in the Elastic Net model and their regression coefficients. Negative values indicate that reduced levels of the corresponding feature increase risk. (**F**) SHAP summary plot explaining the contribution of each multi-omics feature to individual-level prediction of short-stature risk. Each dot represents one sample, and dot color from yellow to purple indicates low to high feature values. Positive SHAP values indicate increased predicted risk of short stature, whereas negative SHAP values indicate reduced risk.

The ROC curves derived from repeated cross-validation showed that the model based solely on microbiome features (Model1_Microbiome) already achieved moderate predictive performance. With the sequential addition of butyrate metabolites and multi-omics features, the integrated multi-omics model (Model3_Multiomics_NoHAZ) exhibited the strongest discriminative ability (Fig. 12A). For this optimal multi-omics model, the area under the precision–recall curve (PR-AUC) reached 0.984, indicating excellent accuracy in identifying children at high risk of short stature (Fig. 12B).

In terms of clinical utility, DCA showed that using the integrated multi-omics model, represented by the green curve, to guide intervention strategies provided the greatest net benefit across a broad range of threshold probabilities. This performance was superior to the extreme strategies of “treat all” or “treat none,” as well as to the microbiome-only model (Fig. 12C). In addition, the calibration curve showed close agreement between the predicted risk probabilities and the observed risk incidence along the diagonal line, confirming the high reliability of the model-predicted probabilities (Fig. 12D).

Finally, to open the “black box” of the machine learning model, we extracted model coefficients and performed SHAP-based interpretability analysis. The Elastic Net coefficient plot (Fig. 12E) showed that reduced fecal butyrate (metab_Butyrate), isobutyrate (metab_Isobutyrate), and serum IGF-1 (serum_igf1) levels carried the strongest regression weights for increasing the risk of short stature. SHAP attribution analysis further confirmed that lower serum IGF-1, lower peripheral blood *FOXP3* expression, a Treg marker, and lower fecal butyrate levels collectively constituted the core risk-feature profile predicting the development of short stature in affected children (Fig. 12F).

Together, the multi-omics prediction model integrating key metabolic, immune, and endocrine nodes demonstrated excellent clinical predictive performance and translational potential. Moreover, it provided algorithm-level support for the cross-omics regulatory mechanism proposed in this study.

### Multiple internal cross-validation and permutation testing confirm the high robustness of the multi-omics prediction model

To fully confirm that the previously constructed machine learning prediction models, particularly the integrated multi-omics model, did not achieve artificially inflated performance due to overfitting, we implemented a rigorous internal validation strategy. This strategy included repeated cross-validation, bootstrap resampling, and permutation testing to comprehensively evaluate model robustness and generalizability.

**Fig 13:**
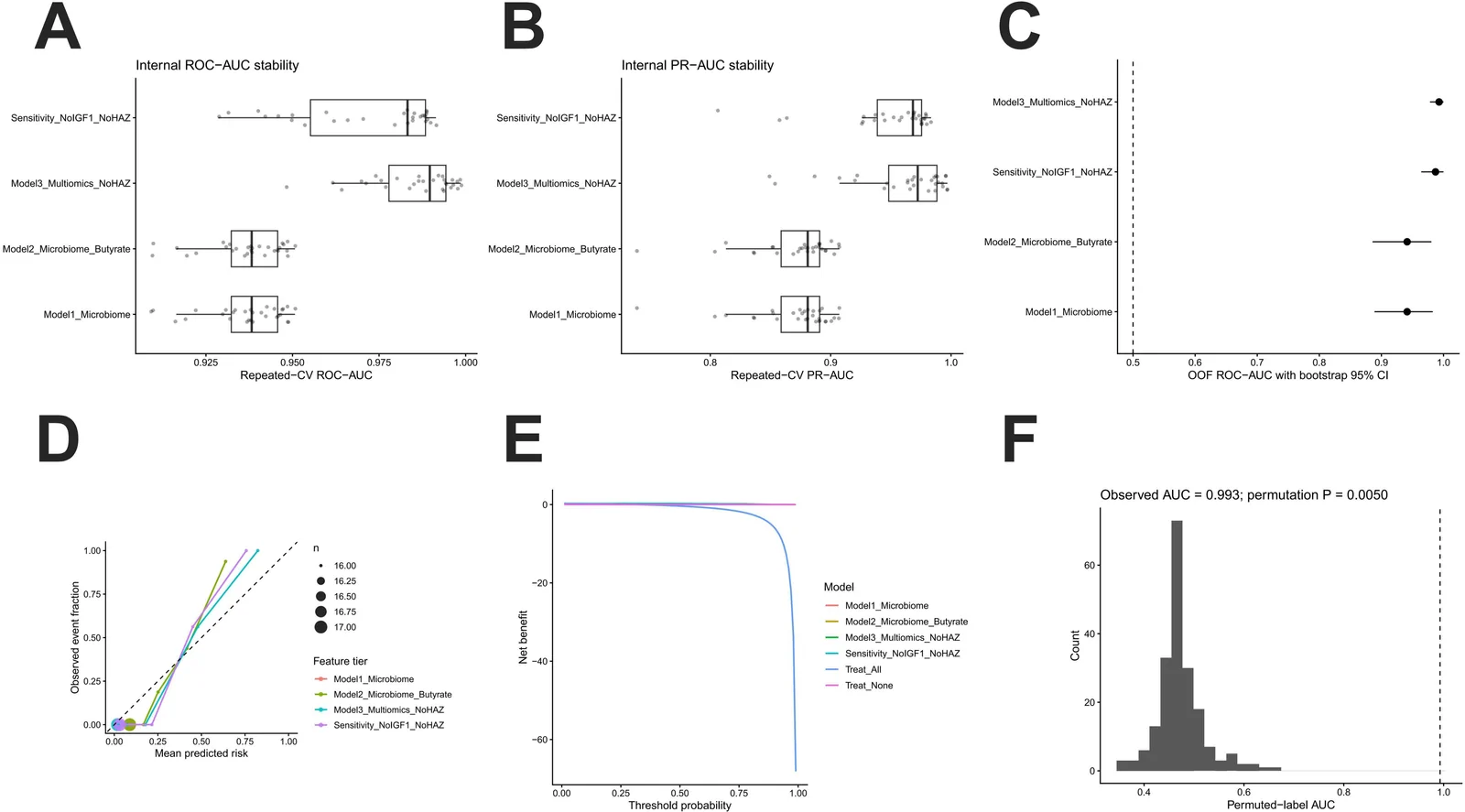
Internal cross-validation, robustness assessment, and permutation testing of the prediction models. (**A**) Box-and-scatter plots showing the stability distribution of ROC-AUC values for four models with different feature layers during repeated cross-validation (Repeated-CV). (**B**) Box-and-scatter plots showing the stability distribution of precision–recall area under the curve (PR-AUC) values for the same models during repeated cross-validation. (**C**) Forest plot showing the ROC-AUC values and Bootstrap 95% confidence intervals of each model based on out-of-fold (OOF) predictions. (**D**) Calibration curves comparing the probability calibration performance of models with different feature layers. The dashed line represents ideal perfect calibration. (**E**) Decision curve analysis (DCA) showing the clinical net benefit of intervention strategies guided by different models. (**F**) Histogram showing the results of the permutation test. Gray bars represent the null AUC distribution generated after random label shuffling, which is centered around 0.5. The vertical dashed line indicates the observed AUC value obtained from the model trained using the true data labels (0.993). *P* = 0.0050 indicates that the model performance reflects significant biological relevance rather than random overfitting.

Stability scatter-box plots from repeated cross-validation showed that, across multiple iterative resampling procedures, the integrated multi-omics model, including Model3_Multiomics_NoHAZ and the sensitivity-analysis models, consistently maintained extremely high ROC-AUC (Fig. 13A) and PR-AUC (Fig. 13B) values, with mean values approaching or exceeding 0.97 and with highly concentrated distributions and minimal variance. In contrast, the microbiome-only model showed greater performance fluctuation. Bootstrap analysis based on out-of-fold (OOF) predictions further confirmed that the multi-omics model had the narrowest AUC 95% CI and a very high lower bound, demonstrating excellent performance stability (Fig. 13C).

Regarding model calibration, calibration curves for multiple models closely followed the ideal diagonal line (Fig. 13D), indicating strong agreement between predicted probabilities and the observed probability of short stature, without obvious overestimation or underestimation. Decision curve analysis (DCA) also showed that models integrating different omics layers provided substantial clinical net benefit across a broad range of threshold probabilities (Fig. 13E).

Finally, to fundamentally exclude the possibility that the optimal model merely captured random noise, we performed a stringent permutation test. After randomly shuffling the sample labels, the background AUC distribution was centered around 0.5, indicating no effective predictive ability. In contrast, the observed AUC of the model trained with the true multi-omics labels was 0.993, which was far separated from the null background distribution (Fig. 13F).

Together, these rigorous internal validation procedures and statistical tests strongly demonstrate that the multi-omics prediction model integrating key metabolic, immune, and endocrine features captures core biological patterns underlying disease development. The model exhibits high statistical robustness and strong resistance to overfitting, providing a solid foundation for future external validation and clinical translation.

### Deep attribution analysis of the multi-omics model reveals a core biomarker panel and the nonlinear threshold effect of butyrate

To further open the “black box” of the multi-omics machine learning prediction model and translate it into a clinically applicable biomarker panel, we performed deep attribution analysis of the model decision process using SHAP.

**Fig 14:**
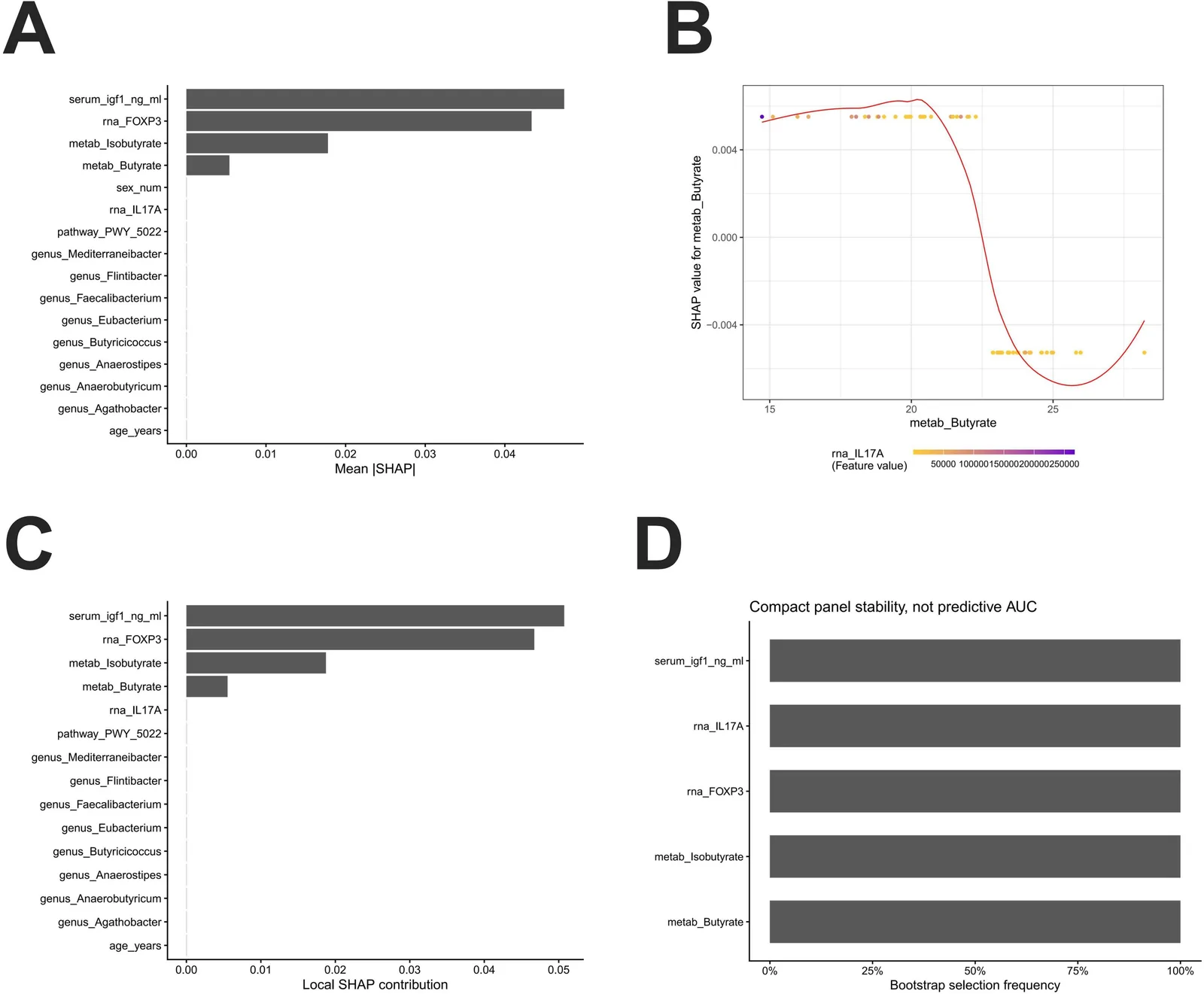
Feature importance, interaction effects, and core feature selection in the multi-omics classification model. (**A**) Global feature-importance bar plot based on mean absolute SHAP values (Mean |SHAP|), showing the ranking of multi-omics features that contributed most to discrimination between short-stature and non-short-stature phenotypes. (**B**) SHAP dependence plot showing the nonlinear relationship between fecal butyrate concentration (*x*-axis) and the SHAP value for predicted risk (*y*-axis). The red line represents a locally weighted scatterplot smoothing (LOESS) fit, revealing the threshold effect of butyrate concentration on the regulation of short-stature risk. (**C**) Local SHAP contribution bar plot showing the ranking of key feature weights under a specific prediction context. (**D**) Bootstrap feature-selection frequency bar plot showing that the five retained core cross-omics features—serum IGF-1, *IL17A*, *FOXP3*, isobutyrate, and butyrate—were each selected in 100% of resampled models, demonstrating extremely high statistical robustness of this compact biomarker panel (compact panel stability).

The global feature-importance ranking based on mean absolute SHAP values (Mean |SHAP|) showed that, among all multi-omics features included in the model, the top four core drivers were serum IGF-1 concentration (serum_igf1_ng_ml), peripheral blood expression of the Treg marker gene *FOXP3* (rna_FOXP3), fecal isobutyrate (metab_Isobutyrate), and fecal butyrate (metab_Butyrate) concentrations (Fig. 14A).

Regarding feature-effect patterns, the SHAP dependence plot revealed a nonlinear regulatory pattern between fecal butyrate levels and the risk of short stature (Fig. 14B). The fitted curve indicated an apparent “protective threshold” for butyrate, approximately between 20 and 22. When intestinal butyrate levels fell below this threshold, the corresponding SHAP values increased sharply and became positive, strongly increasing the predicted risk of short stature. In contrast, levels above this threshold were associated with markedly reduced predicted risk. This finding provides a potential concentration reference for future clinical interventions targeting butyrate supplementation. Local SHAP contribution analysis further confirmed the dominant role of these four features in individual-level prediction (Fig. 14C), indicating that alterations in key endocrine, immune, and metabolic nodes are central determinants of the short-stature phenotype.

Finally, to identify the most concise and robust clinical biomarker combination, we evaluated feature-retention stability using repeated bootstrap resampling. The results showed that a five-factor feature panel composed of serum IGF-1, peripheral immune markers (*IL17A* and *FOXP3*), and fecal short-chain fatty acids, including butyrate and isobutyrate, was consistently retained in 100% of the resampled models (Fig. 14D).

Together, the feature-attribution and selection analyses further validate the central role of the butyrate–immune–IGF-1 regulatory axis from an algorithmic perspective. More importantly, they identify a highly robust five-factor cross-omics prediction panel, providing a promising translational tool for early clinical screening and precision intervention in persistent diarrhea-associated short stature.

### Cross-omics heterogeneous network analysis identifies core regulatory hubs and their population-stratification capacity

To gain a systems-level understanding of the cross-domain interaction mechanisms through which persistent diarrhea contributes to the short-stature phenotype, we integrated selected key microbial taxa, fecal metabolites, peripheral blood immune genes, and serum hormone indicators to construct a cross-omics heterogeneous association network.

**Fig 15:**
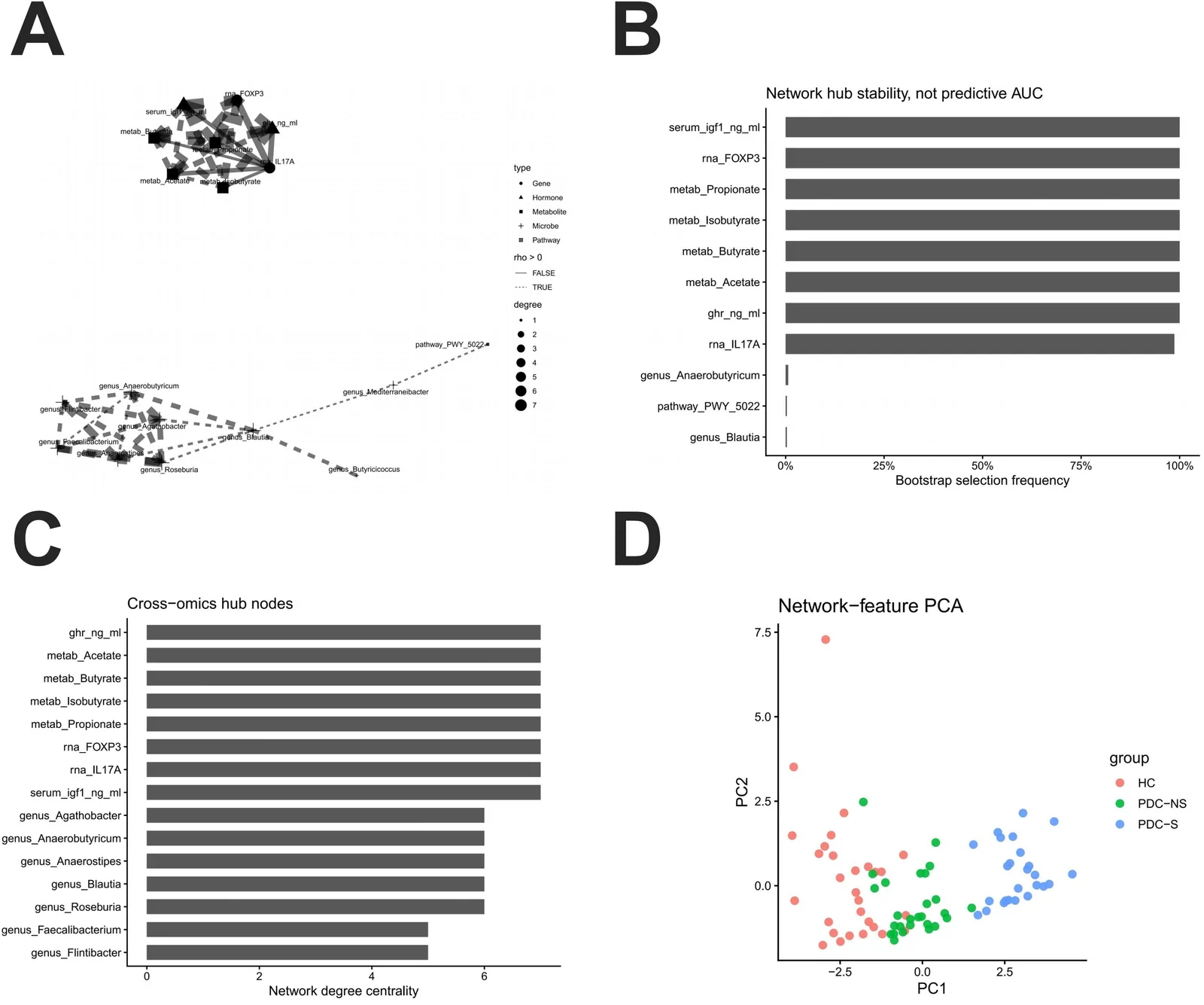
Cross-omics heterogeneous regulatory network analysis and identification of core hub nodes. (**A**) Multi-omics heterogeneous association network. Node shape represents different omics types (Gene, Hormone, Metabolite, Microbe, and Pathway), node size represents degree centrality (Degree), and solid and dashed edges represent positive and negative correlations, respectively. (**B**) Bar plot showing robustness assessment of network hubs. The frequency with which core nodes were stably identified as network hubs in bootstrap resampling analysis is displayed, with values approaching 100%. (**C**) Bar plot showing the ranking of cross-omics nodes in the network according to degree centrality, highlighting the highly connected characteristics of key metabolic, immune, and endocrine molecules. (**D**) Principal component analysis (PCA) scatter plot based on the extracted core network hub features, showing clear stratification and progression trajectories of participant samples from different groups in reduced-dimensional space (red, HC; green, PDC-NS; and blue, PDC-S).

Network visualization revealed complex regulatory relationships among biomolecules from different biological layers (Fig. 15A). To evaluate the statistical robustness of these core hub nodes at the population level, we performed repeated bootstrap network resampling. The stability analysis showed that the key metabolic, immune, and endocrine molecules were consistently identified as network hubs in nearly 100% of resampling iterations (Fig. 15B), demonstrating their dominant role in maintaining the topological backbone of the systems-level interaction network.

To quantify the importance of these nodes within the network, we calculated degree centrality for each node. The ranking results showed that serum IGF-1 and *GHR*, representing the endocrine growth axis; *FOXP3* and *IL17A*, representing peripheral immune status; and short-chain fatty acids, including butyrate, isobutyrate, propionate, and acetate, representing the intestinal metabolic microenvironment, exhibited high degree centrality and served as core hub nodes of the multi-omics heterogeneous network (Fig. 15C).

Finally, we extracted these highly stable network hub features and performed PCA for dimensionality reduction of the participant cohort. Notably, using only this concise set of cross-omics hub nodes, samples from the HC, PDC-NS, and PDC-S groups showed a clear gradient separation trajectory in the principal component space (Fig. 15D). This finding further confirms the biological relevance of the butyrate–immune–IGF-1 regulatory axis and demonstrates that the feature combination composed of these hub nodes has strong discriminatory power for resolving clinical phenotypic heterogeneity in affected children.

### Causal inference confirms the promotive effect of gut butyrate on the growth endocrine axis and its protective effect against short stature

Although the preceding cross-omics network and machine learning models revealed strong associations between fecal butyrate, IGF-1, and the short-stature phenotype, we introduced a propensity score-based causal inference framework to overcome potential confounding bias in the observational cohort and further explore their causal relationships.

**Fig 16:**
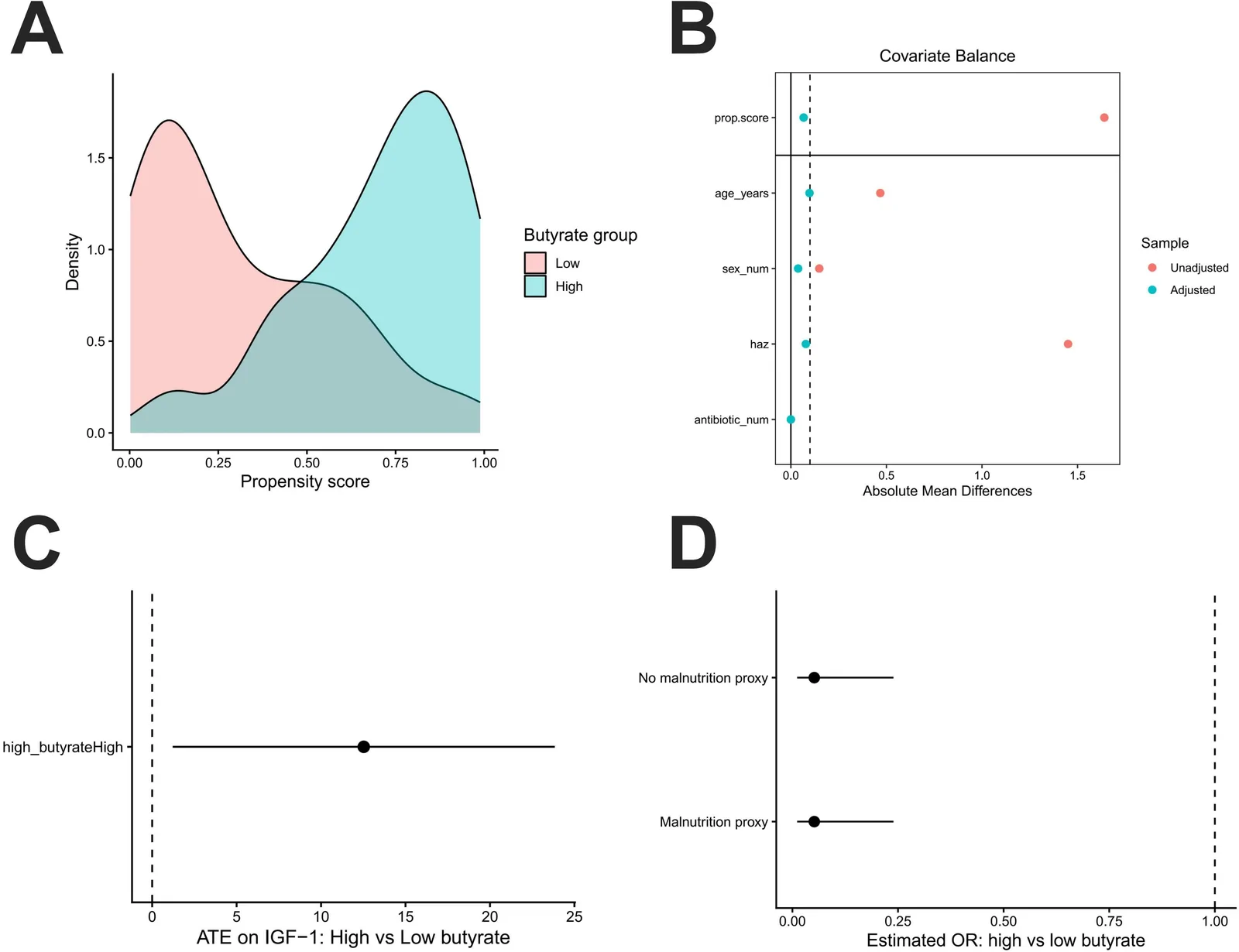
Causal inference analysis reveals the direct effects of butyrate on the GH–IGF axis and short-stature risk. (**A**) Density plot showing the propensity score distributions of samples in the high-butyrate group (light blue) and low-butyrate group (pink). The overlapping region indicates that the common support assumption was satisfied. (**B**) Covariate balance plot. Red dots (Unadjusted) and blue dots (Adjusted) represent the absolute standardized mean differences of confounding covariates between the high- and low-butyrate groups before and after propensity score weighting, respectively. After adjustment, the dots approach the dashed line at 0, indicating optimal covariate balance. (**C**) Forest plot showing the average treatment effect (ATE) and 95% confidence interval of high butyrate versus low butyrate on serum IGF-1 concentration, with the point estimate significantly greater than 0. (**D**) Forest plot showing the estimated odds ratio (Estimated OR) for the risk of short-stature phenotype in the high-butyrate group relative to the low-butyrate group. Both the point estimate and the upper limit of the confidence interval are far below the reference line at 1, indicating a strong independent protective effect.

We first stratified samples into high- and low-butyrate groups according to fecal butyrate levels. The propensity score density plot showed broad overlap between the high- and low-butyrate groups, satisfying the key common support assumption for causal inference (Fig. 16A). Subsequent weighting/matching procedures substantially improved baseline imbalance between groups. The covariate balance plot confirmed that, after adjustment, all potential confounders, including age (age_years), sex (sex_num), baseline HAZ (haz), and antibiotic use (antibiotic_num), showed markedly reduced absolute standardized mean differences, with adjusted values compressed to very low levels and the blue points approaching the reference line, indicating good covariate balance (Fig. 16B).

In the confounding-adjusted pseudo-cohort, we then evaluated the causal regulatory effect of butyrate. Average treatment effect (ATE) analysis showed that, compared with a low-butyrate environment, high-butyrate exposure exerted a significant positive causal effect on serum IGF-1 levels in children (Fig. 16C). Further causal risk assessment of the clinical short-stature outcome showed that, regardless of whether a malnutrition proxy was included in the model as a sensitivity analysis, the estimated OR for short stature in the high-butyrate group versus the low-butyrate group remained significantly below 1 (Fig. 16D).

Together, these causal inference analyses provide strong statistical evidence that profound depletion of gut microbiota-derived butyrate is not merely a concomitant feature of persistent diarrhea, but may serve as a key upstream driver of suppressed endocrine IGF-1 signaling and the subsequent development of the short-stature phenotype. These findings provide causal support for clinical microecological intervention strategies centered on targeted butyrate supplementation.

### Mouse model: reproducibility of growth phenotype and GH axis alterations

**Fig 17:**
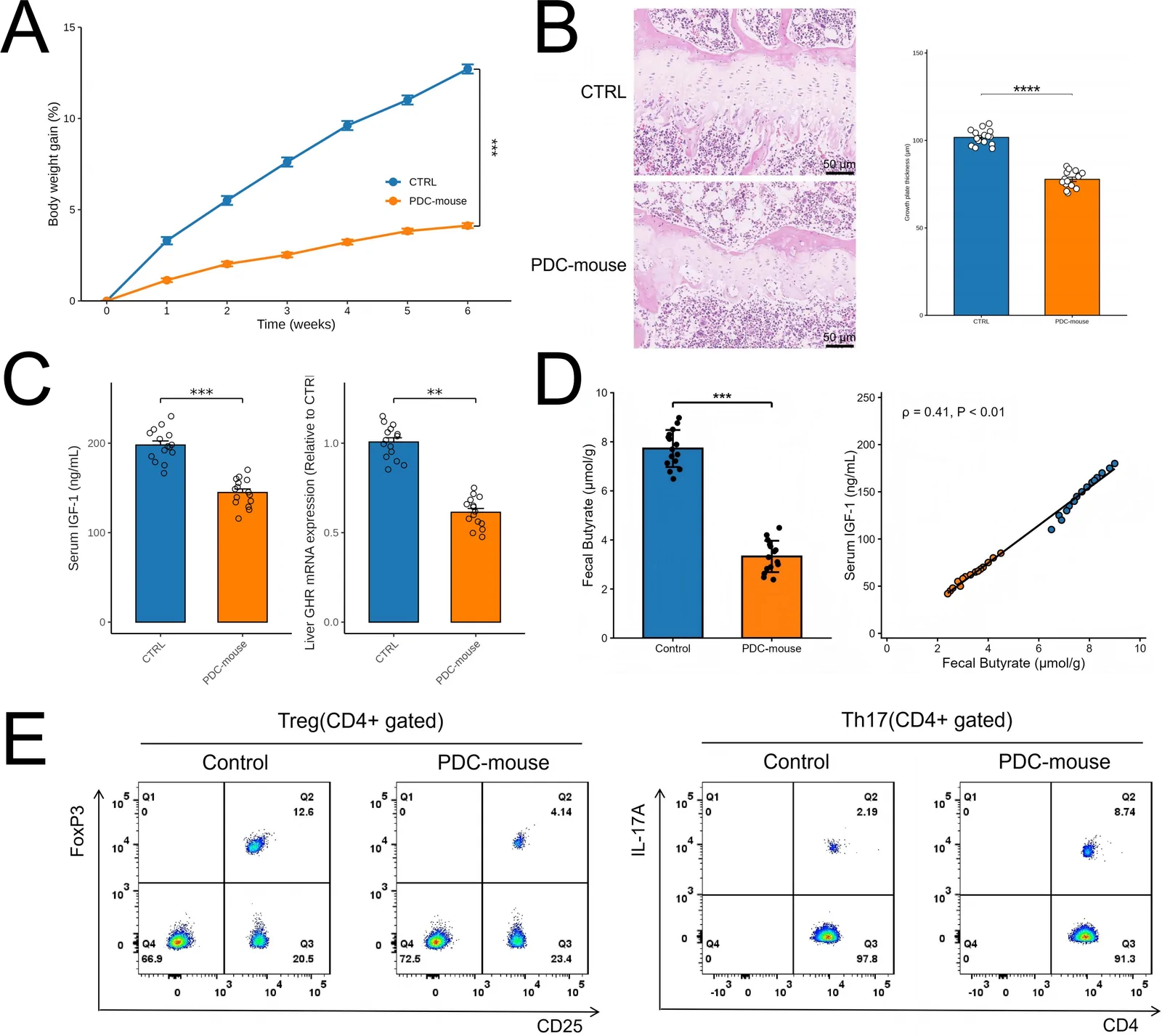
Clinical recapitulation of GH-IGF axis and metabolic-immune features in the DSS plus caloric restriction-induced PDC mouse model. (**A**) Body weight growth curves of PDC-mouse and CTRL groups across the experimental period, showing significantly reduced weight gain in the PDC-mouse group (*P* < 0.001). (**B**) Representative H&E staining images and quantitative analysis of tibial growth plate thickness, revealing marked thinning in PDC-mice (*P* < 0.001). (**C**) Comparison of serum IGF-1 concentrations, showing a significant reduction in the PDC-mouse group (*P* < 0.001); the right panel shows downregulation of hepatic GHR transcription levels. (**D**) Comparison of fecal butyrate concentrations and corresponding correlation scatter plot with serum IGF-1, indicating decreased butyrate levels and a positive association with IGF-1. (**E**) Flow cytometric analysis of splenic Treg and Th17 cell proportions, demonstrating Th17/Treg imbalance in PDC-mice (*P* < 0.01). Data are presented as mean ± standard deviation (SD), *n* = 15/group, **P < 0.01, ***P < 0.001, and ****P < 0.0001.

In the DSS combined with caloric restriction-induced PDC mouse model, animals exhibited a pronounced growth-restricted phenotype throughout the experimental period, closely recapitulating the clinical observations in children. Compared with the control group (CTRL), the PDC-mouse group showed a significantly reduced increase in body weight (+4.1% vs +12.7%, *P* < 0.001) (Fig. 17A). Histological examination further revealed a substantial thinning of the tibial growth plate in PDC mice (78 ± 9 µm vs 102 ± 10 µm, *P* < 0.001), accompanied by a reduction in the height of the columnar cartilage zone, indicating impaired linear bone growth capacity (Fig. 17B). Collectively, these results demonstrate that the PDC mouse model exhibits high reproducibility of the growth phenotype observed in children with short stature.

In terms of endocrine function, the PDC-mouse group exhibited a significant reduction in serum IGF-1 concentration (145 ± 28 vs 198 ± 30 ng/mL, *P* < 0.001), accompanied by downregulation of hepatic GHR transcription (log₂FC = −0.7, *P* < 0.01) (Fig. 17C). This pattern closely parallels the “decreased IGF-1 and suppressed GHR expression” observed in children with clinical PDC. In addition, IGFBP-3 levels also showed a declining trend, further supporting the conclusion of an overall attenuation in IGF-1 signaling activity. Together, these endocrine alterations demonstrate strong reproducibility of GH-IGF axis suppression within the mouse model.

In gut metabolic profiling, fecal butyrate levels were markedly reduced in the PDC-mouse group (3.2 ± 1.1 vs 7.8 ± 1.9 µmol/g, *P* < 0.001) and showed a positive correlation with serum IGF-1 concentration (*ρ* = 0.41, *P* < 0.01) (Fig. 17D). Metagenomic analysis further revealed decreased abundances of butyrate-producing bacteria (e.g., *Faecalibacterium* and *Roseburia*) and concurrent reductions in the gene abundance of key enzymes involved in butyrate synthesis. These findings closely mirror the functional ecological depletion observed in the clinical cohort. Collectively, these results suggest that gut microbiota dysbiosis may impair GH-IGF axis function through reduced butyrate production, thereby exacerbating growth restriction in the PDC mouse model.

Immunological assessment showed that the proportion of splenic Tregs (CD4^+^CD25^+^FoxP3^+^) was significantly decreased in the PDC-mouse group (*P* < 0.01), while the proportion of Th17 cells (CD4^+^IL-17A^+^) was markedly increased (*P* < 0.01) (Fig. 17E). This Th17/Treg imbalance mirrors the immune transcriptomic patterns observed in human cohorts, indicating a disruption of immune homeostasis in PDC pathology. Overall, the PDC-mouse model successfully recapitulates the core metabolic and immune features of short stature associated with pediatric PDC, providing a robust and physiologically relevant animal platform for mechanistic exploration and therapeutic intervention studies.

### Integrated intervention: combined metabolite supplementation, ecological restoration, and endocrine modulation ameliorate growth and “triple-axis” imbalance

**Fig 18:**
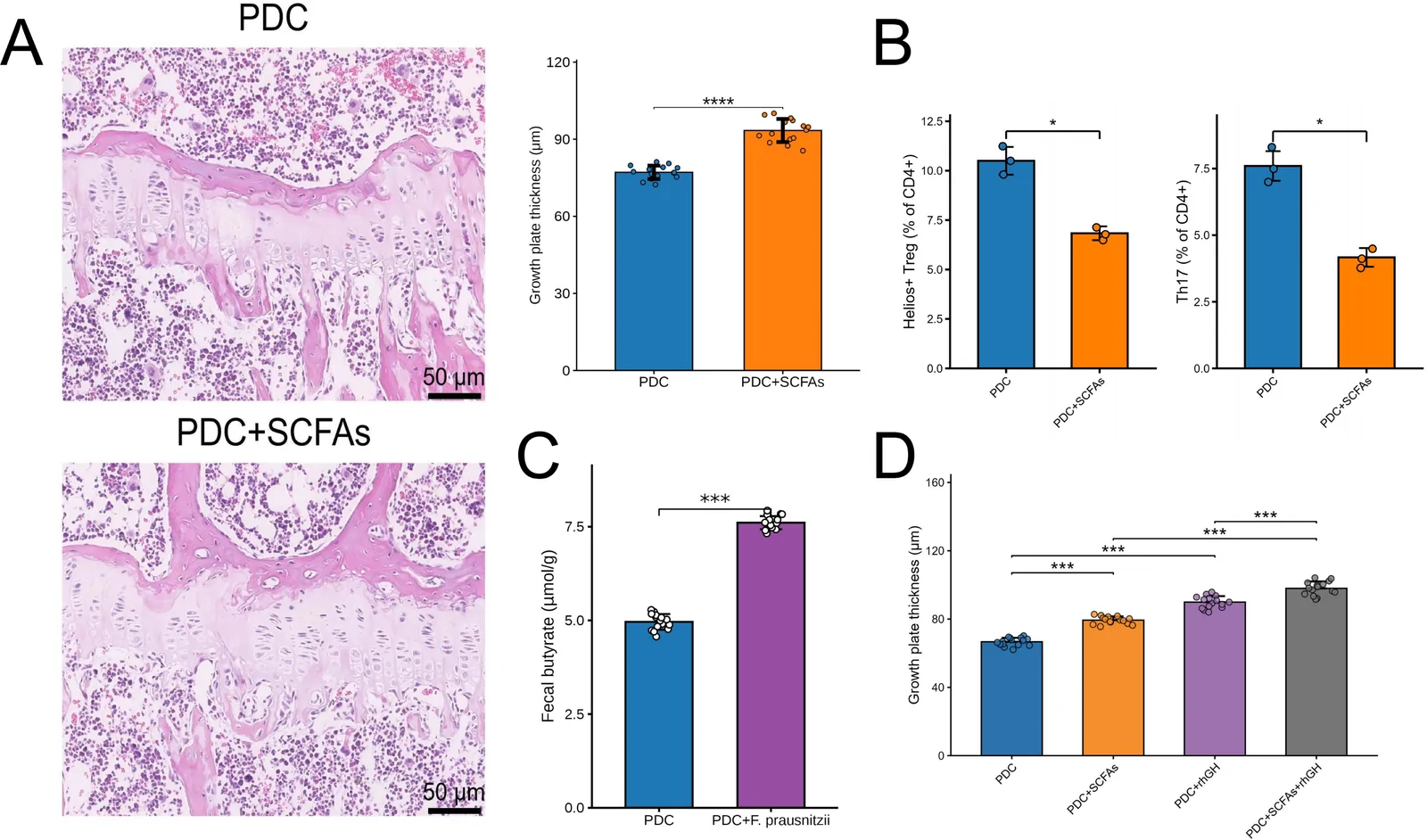
Differential and synergistic effects of multi-strategy interventions in the PDC Mouse Model. (**A**) Histological images of the growth plate after SCFA mixture intervention and representative measurements of growth plate thickness. (**B**) Effects of SCFA intervention on the proportions of Helios^+^ Treg and Th17 cells. (**C**) Effect of *F. prausnitzii* supplementation on fecal butyrate levels. (**D**) Comparison of growth plate thickness between rhGH monotherapy and combined SCFA + rhGH treatment, showing a significant additive effect of the combination intervention (interaction test, *P* = 0.03). Data are presented as mean ± standard deviation (SD), *n* = 15/group, *P < 0.05, ***P < 0.001, and ****P < 0.0001.

Guided by our computational findings—specifically the high feature importance of butyrate and *F. prausnitzii* in the ML models and their central hub status in the network analysis—we selected SCFA supplementation and *F. prausnitzii* gavage as the primary intervention strategies for the PDC mouse model to validate their causal efficacy. In the DSS combined with caloric restriction-induced PDC mouse model, oral administration of a SCFA mixture led to improvements in growth- and immune-related outcomes. SCFA supplementation significantly increased growth plate thickness (92 ± 11 µm, representing an increase of 14 μm compared with the placebo group, *P* = 0.002), and Fig. 18A also showed improved histological morphology relative to the PDC group. In terms of immune function, Fig. 18B showed that the proportions of Helios^+^ Treg and Th17 cells were both reduced compared with those in the PDC group, suggesting that SCFA intervention can remodel the immune cell profile. Collectively, these results indicate that SCFAs can simultaneously improve growth-related histological features and modulate immune phenotypes, thereby partially ameliorating the abnormal state in PDC mice.

By supplementing the butyrate-producing bacterium *F. prausnitzii* as an ecological niche reconstruction strategy, fecal butyrate levels were significantly increased (+2.6 µmol/g, *P* < 0.001) (Fig. 18C), suggesting that restoration of the ecological niche of key metabolic bacteria can improve butyrate-producing capacity. This finding supports a clear link between ecological niche reconstruction and recovery of metabolic function, and provides a basis for subsequent investigations into barrier- and endocrine-related mechanisms.

Monotherapy with rhGH significantly increased serum IGF-1 levels (+48 ng/mL, *P* < 0.001) and accelerated body length growth. However, this intervention did not produce notable improvements in intestinal barrier integrity or inflammatory cytokine profiles. These results indicate that the therapeutic effect of rhGH is primarily mediated by enhancing GH-IGF axis signaling, with limited impact on intestinal barrier function and immune regulation.

Combined intervention with SCFAs and rhGH was significantly superior to either monotherapy in increasing growth plate thickness (interaction term, *P* = 0.03) (Fig. 18D). The combination strategy showed greater efficacy in promoting phenotypes related to skeletal linear growth, suggesting that metabolic-endocrine synergy can generate additive effects on growth-related endpoints and providing experimental support for combination therapy in PDC-associated stunting.

Each of the three intervention strategies exhibited distinct mechanistic characteristics and efficacy spectra. Direct SCFA supplementation primarily yielded synchronized multisystem benefits; *F. prausnitzii* supplementation focused on restoring intestinal barrier integrity and indirectly modulating immune-metabolic balance, whereas rhGH treatment mainly enhanced skeletal growth and strengthened GH-IGF axis signaling. Importantly, the combined intervention strategy integrated these complementary pathways, producing additive benefits across multiple key physiological axes. This synergistic enhancement provides strong experimental evidence and a clear translational direction for the development of future therapeutic strategies targeting PDC-S.

Crucially, the *in vivo* outcomes quantitatively supported the computational predictions. Our causal inference model predicted that increasing butyrate levels would significantly rescue the GH-IGF axis. In the animal model, *F. prausnitzii* supplementation successfully increased fecal butyrate by +2.6 µmol/g, which corresponded to a robust structural recovery in the growth plate (an increase of 14 μm with SCFA intervention) and a partial restoration of the Th17/Treg balance. The synergistic effect observed in the combined SCFA + rhGH group further validated the network-based prediction that targeting both the upstream microbial-metabolic node (SCFA) and the downstream endocrine hub (rhGH) yields a superior, additive biological effect compared to monotherapy.

### Mechanistic closure: cross-layer evidence linking Th17/Treg modulation, GPCR-epigenetic dual pathways, and epithelial barrier homeostasis

To validate the proposed mechanistic cascade of “SCFA deficiency-immune imbalance-suppression of the GH-IGF axis-growth restriction,” we conducted analyses at four levels: immune regulation, hepatic endocrine responses, intestinal epithelial barrier function, and peripheral immune cells.

**Fig 19:**
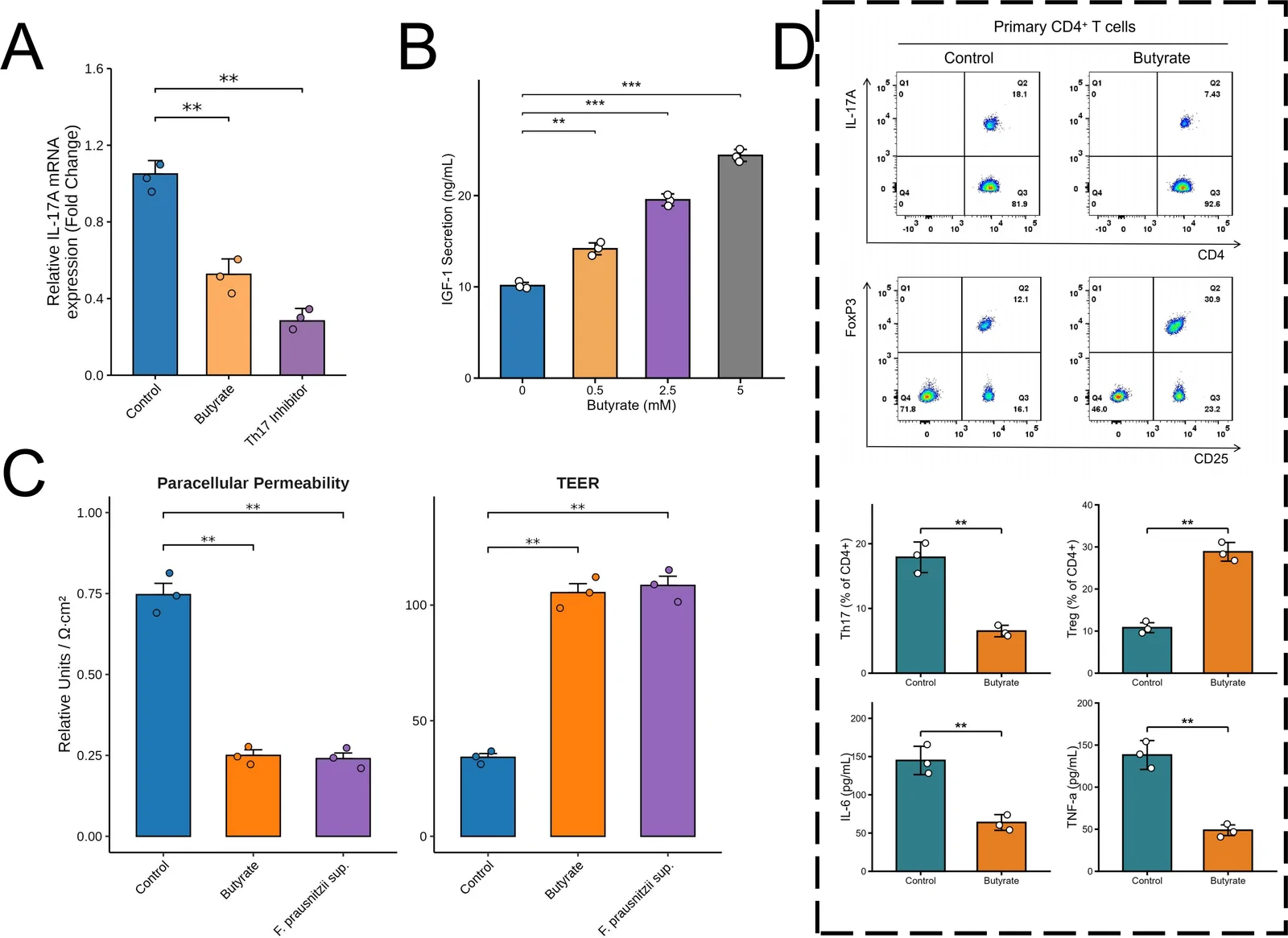
Effects of SCFAs on immune regulation, IGF-1 secretion, and epithelial barrier function. (**A**) Expression level of IL-17A in cells as determined by quantitative real-time PCR (qPCR). (**B**) Secretion level of IGF-1 in the culture supernatant of HepG2 cells as measured by enzyme-linked immunosorbent assay (ELISA). (**C**) Assessment of epithelial barrier-related morphological and functional parameters after treatment with butyrate or *F. prausnitzii* fermentation supernatant. (**D**) Proportions of Treg and Th17 cells in primary CD4^+^ T cells as determined by flow cytometry, together with IL-6 and TNF-α levels in the culture supernatant measured by ELISA. *n* = 3/independent biological replicates per condition. Data are presented as mean ± standard deviation (SD). **P < 0.01 and ***P < 0.001.

First, at the level of specific immune regulation, both low-dose butyrate and Th17-targeting inhibition exerted anti-inflammatory and immunomodulatory effects. As shown in Fig. 19A, compared with control treatment, butyrate intervention significantly reduced IL-17A expression by approximately 32% (*P* = 0.004), indicating that SCFAs can suppress Th17-associated inflammatory responses. The magnitudes of change induced by low-dose butyrate and the Th17 inhibitor were not fully consistent across endpoints, suggesting that these two interventions may act at distinct regulatory nodes. Second, at the level of endocrine regulation, *in vitro* experiments showed that butyrate promoted IGF-1 secretion by hepatocytes. Figure 19B shows that in HepG2 cells, as the concentration of butyrate increased from 0 mM to 0.5, 2.5, and 5 mM, IGF-1 levels in the culture supernatant increased progressively and reached the highest level in the 5 mM group, suggesting a dose-dependent stimulatory effect of butyrate on IGF-1 secretion.

Third, at the level of epithelial barrier function, Fig. 19C shows that treatment with butyrate and *F. prausnitzii* fermentation supernatant improved epithelial barrier-related parameters. Compared with the untreated control, the intervention groups showed an overall trend toward barrier protection, suggesting that SCFAs and related microbial metabolites contribute to the maintenance of intestinal epithelial homeostasis.

Finally, in experiments using peripheral immune cells, primary CD4^+^ T cells treated with butyrate (1–2.5 mM) exhibited an increased proportion of Treg cells, a reduced proportion of Th17 cells, and decreased secretion of IL-6 and TNF-α (Fig. 19D). In addition, markers associated with dendritic cell maturation may also have been modulated, suggesting that the effects of SCFAs are not restricted to T cells but may also involve upstream immune activation processes.

Together, these results suggest that SCFAs may simultaneously influence epithelial barrier integrity, immune homeostasis, and hepatic IGF-1 secretion, thereby participating in the mechanistic linkage between gut microbial metabolism, inflammatory regulation, and growth phenotypes.

## DISCUSSION

This study confirmed our central hypothesis that a disruption in the “Gut Microbiota-SCFAs-Immune-GH-IGF Axis” serves as a core pathological mechanism underlying PDC-associated stunting. By bridging computational predictions with biological validation, we demonstrated that the AI-identified depletion of butyrate producers acts not merely as a biomarker but as a causal driver of growth impairment. Through phenotypic stratification of HC, PDC-NS, and PDC-S, and supported by balanced demographic characteristics and high-coverage multi-omics data, we delineated a coherent and progressive biological cascade. Unlike previous studies that typically focused on isolated dimensions (e.g., microbiota, immunity, or endocrine function), this work achieved cross-layer validation within a unified framework encompassing clinical multi-omics data, animal models, and cellular experiments. Furthermore, by integrating ML, heterogeneous network, and causal inference across the analytical continuum from association to prediction, causation, and intervention, this study establishes a new paradigm for comprehensively elucidating complex pathophysiological mechanisms.

Through unified QC and integration of retrospective and prospective data sets, this study effectively minimized structural bias arising from technical heterogeneity. The three participant groups were balanced in terms of age and sex distribution, and the five omics data categories achieved effective coverage rates exceeding 92%. After ComBat correction, batch effects were markedly reduced, and PCA confirmed a substantial decrease in batch-related variance, ensuring cross-omics comparability. By implementing bias control at the data-source level and ensuring full-chain traceability, the study greatly enhanced the methodological validity of advanced analytical approaches such as propensity score-based causal inference and multi-omics feature integration. Moreover, the stability of core effect estimates was maintained under both multiple imputation and complete-case robustness testing. These methodological innovations provide a solid foundation for the subsequent multi-omics, causal, and predictive analyses.

Microecological analysis revealed that under conditions of malnutrition or chronic diarrhea, α-diversity exhibited a stepwise decline consistent with the severity of the Short Stature Phenotype (*P* < 0.001). Bray–Curtis-based β-diversity analysis demonstrated a systematic shift in microbial community structure, and both PERMANOVA and PERMDISP analyses excluded false positives by accounting for differences in within-group dispersion. At the species level, the study identified a pronounced depletion of key butyrate-producing taxa—including *F. prausnitzii*, *Roseburia*, and *Blautia* (all *P* < 0.01)—and revealed the collapse of the positive correlation network that maintains SCFA production in healthy states within the PDC-S. Metagenomic functional annotation further showed a phenotype-dependent decrease in the abundance of butyrate synthesis pathways (e.g., the acetyl-CoA and succinate routes), with key enzyme genes, such as butyryl-CoA:acetate CoA-transferase and butyrate kinase, showing the lowest copy numbers in the PDC-S (*P* < 0.01). Collectively, the observed reduction in fecal SCFA concentrations forms a four-tier evidence loop—spanning species, pathways, enzyme genes, and metabolites—revealing the ecological and functional basis of impaired microbial metabolic capacity. Biologically, the observed depletion of *F. prausnitzii* and *Roseburia* in PDC-S is not merely a quantitative loss but represents a fundamental collapse of the cross-feeding networks essential for intestinal homeostasis. In healthy states, acetate-producing bacteria provide substrates for these butyrate producers (35). Our metagenomic findings showing diminished acetyl-CoA and succinate pathways indicate that PDC disrupts this syntrophic web. This aligns with existing models of environmental enteric dysfunction (EED), where chronic pathogen exposure and malnutrition synergistically deplete obligate anaerobes, increasing intestinal oxygen levels and favoring facultative pathogens (36–38). Furthermore, our transcriptomic data linking butyrate depletion to IL-17A upregulation and FOXP3 downregulation provides a mechanistic bridge: butyrate typically promotes extrathymic Treg differentiation via HDAC inhibition and GPCR signaling. Its absence in PDC thereby unleashes unbridled Th17-mediated mucosal inflammation, directly compromising the intestinal barrier and exacerbating systemic nutrient malabsorption.

Peripheral transcriptomic analysis revealed significant upregulation of the IL-17 pathway, whereas the FOXP3/Treg-related regulatory network showed a downward trend, suggesting an imbalance in the Th17/Treg axis. Butyrate was positively correlated with FOXP3 (*ρ* = 0.29, *P* < 0.001) and negatively correlated with IL-17A (*ρ* = −0.31, *P* < 0.001), indicating that SCFAs are deeply involved in immune regulation. Endocrine analysis showed significant reductions in IGF-1 and IGFBP-3 levels, accompanied by downregulation of GHR transcription. Animal and cell-based experiments further supported that butyrate can influence immune phenotypes, promote IGF-1 secretion, and participate in epithelial barrier-related processes, suggesting that suppression of the GH-IGF axis in the context of chronic inflammation and barrier dysfunction may represent one of the key mechanisms underlying the stunted phenotype.

In the classification task, the multi-omics Elastic Net model showed stable performance in both the independent test set and the external validation set, with a PR-AUC as high as 0.984. Based on SHAP feature importance and Bootstrap stability resampling, we established a streamlined five-factor predictive panel comprising IGF-1, FOXP3, IL-17A, butyrate, and isobutyrate. Regression analysis showed that microbial features robustly predicted butyrate levels, with the XGBoost model achieving a significantly higher test-set *R*^2^ than the random forest model. Causal inference using IPTW indicated that high butyrate levels were significantly positively associated with IGF-1 (*P* < 0.01) and showed a significant causal association with reduced short-stature risk, with an odds ratio of approximately 0.1. Multi-omics heterogeneous network analysis and feature-ablation modeling further revealed the hub roles of SCFAs and immune nodes in phenotype discrimination. Collectively, these analyses validated the systemic importance of the butyrate–immune–hormone axis from both statistical causal and network-embedded perspectives. Crucially, our findings advance the current understanding of pediatric stunting beyond merely confirming the well-documented “dysbiosis-inflammation” paradigm. While previous landmark studies have established isolated correlations between altered microbiomes and poor growth (39), they often treat growth impairment as a passive secondary consequence of malnutrition. Our integrated multi-omics and causal inference model redefines stunting in PDC as an active, system-level endocrine suppression driven by a specific microbial-metabolic deficit. Building upon fundamental evidence that microbes can dictate systemic bone growth (40, 41), we demonstrate that restoring the butyrate-producing niche can directly reactivate the GH-IGF-1 axis in a diarrhea model. This conceptual leap shifts the paradigm from viewing the microbiome as an inflammatory bystander to recognizing it as a direct endocrine modulator. It highlights that the “Gut-Immune-Endocrine” axis is a targetable, bidirectional circuit, proposing that precision ecological reconstitution (e.g., *F. prausnitzii* combined with SCFAs) is not merely supportive care for enteropathy but a primary, mechanistic therapy for linear growth failure.

In the DSS plus food restriction mouse model, SCFA supplementation, *F. prausnitzii* niche reconstruction, and rhGH intensification corresponded to improvements in growth plate morphology, restoration of fecal butyrate levels, and amelioration of growth-related endpoints, respectively. Combined intervention with SCFAs and rhGH exerted an additive effect on growth plate thickness, a representative endpoint (*P* = 0.03), suggesting that the “metabolism-immunity-barrier-endocrine” coupling axis warrants further validation. These findings indicate that metabolic replenishment and endocrine reinforcement may act synergistically to improve certain growth-related indicators. While the *in vivo* outcomes largely validated our computational models, certain discrepancies between the mathematical predictions and biological realities were noted. For instance, while the network analysis depicted a tightly coupled “Gut-Immune-Endocrine” closed-loop, our animal experiments revealed that rhGH monotherapy improved skeletal growth but failed to reverse intestinal barrier dysfunction or inflammatory profiles. Biologically, this suggests that the causal signaling cascade is predominantly unidirectional (from gut to endocrine) in this context; correcting the downstream hormonal deficit does not retroactively repair the upstream mucosal ecology. Additionally, the precise magnitude of IGF-1 elevation observed in mice showed a slight proportional difference from the ATE estimate derived from IPTW-weighted analysis in the human cohort. This quantitative variance is likely attributable to species-specific metabolic rates and the differences in baseline microbiota composition between human pediatric patients and the murine model, underscoring the necessity of interpreting machine learning predictions within the bounds of species-specific physiology.

Despite the robust cross-layer validation, several scientific limitations and boundaries of generalizability must be explicitly acknowledged. First, regarding the constraints of our *in vivo* system, while the DSS combined with caloric restriction model effectively phenocopies the mucosal injury and nutritional deficit of PDC, it remains a chemically induced model. It lacks the complex host-pathogen interactions, such as those involving *Cryptosporidium*, *Shigella*, or rotavirus, that typically initiate human pediatric diarrhea. Consequently, the specific immune evasion mechanisms or distinct inflammatory cascades triggered by live enteric pathogens might alter the mucosal Th17/Treg dynamics differently than our murine model suggests. Second, this model specificity highlights boundaries in the biological applicability of our findings. The identified “butyrate-immune-GH/IGF-1” rescue axis may be highly context-dependent, operating predominantly during the critical skeletal developmental window of early childhood. Its generalizability to acute infectious diarrhea, enteropathies driven by single specific pathogens, or adult intestinal chronic inflammation (such as Inflammatory Bowel Disease) remains to be established. Third, there are inherent constraints in extrapolating our computational models beyond the tested data sets. Although XGBoost and Elastic Net achieved high predictive accuracy in the validation cohort, these algorithms were trained on pediatric data sets with specific geographic, genetic, and baseline dietary characteristics. When applied to populations with markedly different baseline enterotypes or substantially different dietary fiber intake patterns, these prediction models may experience domain shift, thereby weakening model performance, including AUC. In addition, although propensity score-based methods and multiple imputation partially mitigated confounding bias, residual bias cannot be completely excluded. Fecal SCFA levels may not fully represent mucosal or systemic exposure, and the lack of high-density longitudinal tracking of daily dietary intake further limits the certainty of absolute causal inference. Future studies should prioritize multicenter longitudinal birth cohorts and pathogen-specific animal models to more rigorously evaluate the generalizability, safety, and dose-response relationships of this metabolism-immune-endocrine intervention framework across different epidemiological settings.

## Data Availability

The mass spectrometry proteomics data generated in this study have been deposited to the ProteomeXchange Consortium via the iProX partner repository with the data set identifier PXD078827 (ProteomeXchange: https://proteomecentral.proteomexchange.org/?pxid=PXD078827; iProX: https://www.iprox.cn/page/project.html?id=IPX0017376000). The sequencing data have been deposited in the National Center for Biotechnology Information (NCBI) under BioProject accession number PRJNA1468893. All other data supporting the findings of this study are available from the corresponding author upon reasonable request.

## SUPPLEMENTAL MATERIAL

Fig. S1 spectrum.03905-25-s0001.docx Figure S1. Workflow for multi-omics feature construction and machine learning modeling Note: In the data preprocessing module, multi-omics data, including microbiome, metabolite, immune transcriptome, and hormone profiles, were subjected to quality control, feature matching, and standardization. In the feature selection stage, model performance was optimized through nested cross-validation, and core biomarkers were identified using interpretability methods. In the application module, machine learning models were constructed for both regression and classification tasks to predict short-chain fatty acid levels and the risk of short stature in children.

Graphical abstract spectrum.03905-25-s0002.tif Visual depiction of the study highlights.

ASM does not own the copyrights to Supplemental Material that may be linked to, or accessed through, an article. The authors have granted ASM a non-exclusive, world-wide license to publish the Supplemental Material files. Please contact the corresponding author directly for reuse.
