# Supplementary material for: Multi-omics insights into growth impairment mechanisms in children with persistent diarrhea
Source: Microbiol Spectr. 2026 Sep 17;14(10):e03905-25. doi: 10.1128/spectrum.03905-25 (PMC13689471; doi:10.1128/spectrum.03905-25)
Supplement: Supplementary file 1 [file spectrum.03905-25-s0001.docx]

**Figure S1. Workflow for multi-omics feature construction and machine learning modeling**

Note: In the data preprocessing module, multi-omics data, including microbiome, metabolite, immune transcriptome, and hormone profiles, were subjected to quality control, feature matching, and standardization. In the feature selection stage, model performance was optimized through nested cross-validation, and core biomarkers were identified using interpretability methods. In the application module, machine learning models were constructed for both regression and classification tasks to predict short-chain fatty acid levels and the risk of short stature in children.
